# The auditory nerve glial transition zone is a focal site of age-related immune-myelin interactions

**DOI:** 10.3389/fimmu.2026.1757234

**Published:** 2026-08-07

**Authors:** Shelby A. Payne, Haley R. Anderson, Isaac M. Brown, Jiaxin Chai, Peng Chen, Hai Yao, Jeremy L. Barth, Hainan Lang

**Affiliations:** 1 Department of Pathology and Laboratory Medicine, Medical University of South Carolina, Charleston, SC, United States; 2 Clemson-MUSC Bioengineering Program, Department of Bioengineering, Clemson University, Clemson, SC, United States; 3 Department of Oral Health Sciences, Medical University of South Carolina, Charleston, SC, United States; 4 Department of Regenerative Medicine and Cell Biology, Medical University of South Carolina, Charleston, SC, United States; 5 Ralph H. Johnson Department of Veterans Affairs Medical Center, Charleston, SC, United States

**Keywords:** age-related hearing loss, aging, auditory nerve degeneration, cochlear macrophage, demyelination, glial transition zone, microglia

## Abstract

The auditory nerve (AN) glial transition zone (GTZ), where myelination shifts from Schwann cells in the peripheral AN to oligodendrocytes in the central AN, is a unique biological niche housed within the cochlear modiolus. Although age-related hearing loss (ARHL) is associated with AN degeneration, the neuroimmune mechanisms contributing to age-related myelin pathology and altered immune cell function remain poorly defined. We hypothesized that the AN GTZ is especially vulnerable to age-related immune activity which may contribute to AN degeneration and myelin pathology. Using an ARHL mouse model, we combined large-field light sheet microscopy of tissue-cleared mouse temporal bones, three-dimensional high-resolution imaging, and quantitative immunohistochemistry to evaluate the AN GTZ. RNA-sequencing was used to evaluate age-related changes in gene expression of the whole AN. Human temporal bones were also examined to evaluate the AN and its macrophages/microglia. RNA-sequencing revealed age-associated enrichment of neuroinflammation and myelin degeneration-related pathways. Aging was associated with an increase in Iba1^+^ macrophages/microglia throughout the AN, with prominent accumulation at the AN GTZ. The aged AN GTZ contained Iba1^+^ subpopulations expressing CD206, Gal3, CD163, and Lamp1, suggesting enhanced phagocytic activity and altered activation state at the AN GTZ. CD68^+^ cells were also increased at the AN GTZ, as well as the central AN, with aging. Macrophages/microglia contained significantly more internalized myelin debris across all regions of the AN with aging, including myelin localized within Lamp1^+^ lysosomes of Iba1^+^ cells. More importantly, normal-appearing myelin was frequently observed within macrophages/microglia at the AN GTZ, but not in the peripheral or central regions of the AN. Myelin debris within Iba1^+^ cells was also found in the human AN, consistent with our findings in mouse. Together, these findings identify the AN GTZ as a unique microenvironment with age-related alterations in phagocytic activity and immune activation. The immune cell heterogeneity and enhanced myelin phagocytosis observed in the aging AN emphasize the importance of defining how distinct macrophage/microglia populations contribute to AN degeneration and ARHL.

## 1 Introduction

Two-thirds of adults over the age of 65 years old are affected by age-related hearing loss (ARHL) (1). Although there are numerous contributors to the pathology of ARHL, age-related changes in immune cell activity remain understudied. The auditory nerve (AN), which is one branch of cranial nerve VIII (vestibulocochlear nerve), conducts sound from the cochlea to the brainstem (2). The AN contains a diverse population of immune cells and myelinating glia (3). The peripheral AN is myelinated by Schwann cells, while the central AN is myelinated by oligodendrocytes (4, 5). The region where myelination shifts from Schwann cells to oligodendrocytes is called the glial transition zone (GTZ) (i.e., Obersteiner-Redlich zone) and creates a unique biological niche due to the close proximity of peripheral and central myelinating glia (6). While such central-peripheral interfaces have been defined in other cranial nerves and the spinal cord, the GTZ is understudied in the auditory system (7–11). The GTZ of the AN is unique because it integrates the peripheral and central glial microenvironments within the bony confines of the cochlea.

Previous studies in the spinal cord suggest that such central-peripheral transition zones serve as important immune interfaces that regulate myelin turnover during development and after injury (6, 12–14). Within the brain, both resident microglia and peripheral macrophages play important roles in myelination during development and myelin maintenance, consistent with the dynamic nature of myelination throughout an individual’s lifetime (15–18). However, in the aging nervous system, these processes become dysregulated, leading to myelin breakdown, chronic inflammation, and axonal degeneration (19–25). Myelin is critical to AN function because it preserves temporal synchrony required for speech perception (5, 26–28). However, the effects of aging on macrophages/microglia and their interactions with myelin at the AN GTZ remain undefined.

The dynamics of the AN immune cell compartment and associated changes to myelination within the aging AN are critical for determining the complex mechanisms of ARHL and other cochlear pathologies. Although prior studies have described macrophages in the cochlea during development, aging, and acute noise injury (24, 29–32), the effects of aging on the immune cells that occupy the AN remain largely unexplored. Furthermore, the extent to which these age-related changes differ between the peripheral AN, the central AN, and the AN GTZ has not been addressed in animal models or in human temporal bones.

The goal of this study is to define aging effects on AN myelination and immune activity across distinct regions of the AN. Utilizing large-field light sheet microscopy of tissue-cleared mouse temporal bones, three-dimensional high-resolution imaging, quantitative immunohistochemistry, and RNA-sequencing in an established ARHL mouse model, we examined aging effects on the AN GTZ, macrophage/microglia populations, myelination, and phagocytic activity within the peripheral AN, the central AN, and the AN GTZ. We also assessed the peripheral AN, central AN, and AN GTZ in human temporal bones.

## 2 Materials and methods

### 2.1 Animals

All studies were performed in accordance with the guidelines of the Institutional Animal Care and Use Committee of the Medical University of South Carolina (MUSC) and the Ralph H. Johnson Veterans Affairs Health Care System (VAHCS) in Charleston, South Carolina. Breeding pairs for CBA/CaJ mice were originally purchased from The Jackson Laboratory (JAX#000654) and bred at the MUSC Animal Research Facility. Mice were bred and housed in a low-noise vivarium with a 12/12-hour light/dark cycle and given standard lab chow and water ad libitum. Young (1.5–4 months) and aged (>2.1 years) CBA/CaJ mice (of both sexes) were used in this study. The number of mice per group is reported in the figure legends. Mice with signs of external ear canal obstruction, middle ear obstruction, or infection were excluded.

### 2.2 Assessment of auditory function

All mice used in this study underwent auditory brainstem recordings (ABR) as previously described (33–35). Mice were anesthetized with an intraperitoneal injection (10 mg/kg xylazine and 100 mg/kg ketamine). ABRs were performed in an acoustically isolated booth (IAC Acoustics), and mice were placed on a 37 °C heating pad, with artificial tear ointment on both eyes. Subdermal needle electrodes (F-EZ-24, Genuine Grass Reusable Subdermal Needle Electrodes) were placed on the vertex (recording), ipsilateral mastoid (reference), and hind limb (ground). ABRs were digitized at 15 kHz using a low-impedance head stage connected to a pre-amplifier (RA4LI/RA4PA, Tucker Davis Technologies). Electrode impedance was checked at the start of each session to ensure it did not surpass 3 kΩ. The pre-amplifier was connected to an RZ6 input/output device (Tucker Davis Technologies) and responses were recorded with BioSigRZ software (Tucker Davis Technologies). Audiometric thresholds were evaluated with 1.1 ms tone pips with 0.55 ms cosine rise-fall time presented from 90 dB SPL to 5 dB SPL using 5 dB steps. Stimuli were presented in a closed field through an MF1 speaker (Tucker Davis Technologies) coupled to a 3 mm diameter plastic tube and earpiece inserted into the ear canal. Presentation rate of stimuli was 21 Hz with an average of 512 repetitions. ABR Wave I thresholds at 11.3 kHz were determined via the lowest stimulus level with an identifiable and reproducible Wave I. Aged mice had elevated thresholds compared to young mice in agreement with reported auditory functional declines in this mouse strain by our laboratory and others (24, 36–38) (**Supplementary Figure 1**).

### 2.3 Mouse cochlear tissue collection and preparation

Mice were euthanized and transcardial perfusion was performed with 4% paraformaldehyde (Electron Microscopy Sciences) solution in 1x phosphate buffered saline, pH 7.4 (1x PBS). Temporal bones were then collected, immediately bath-fixed at 4 °C with 4% paraformaldehyde solution in 1x PBS, then perfused via the round and oval windows. Temporal bones were kept in fixative for 24 hours at 4 °C. Fixed cochleae were then decalcified in 10% ethylenediaminetetraacetic acid (EDTA) for 1–3 days depending on age of the mouse. Each cochlea was then cryoprotected in a 15%/20%/30% sucrose gradient over 2 days. After cryoprotection, cochleae were embedded in Tissue-Tek OCT compound and sectioned at a thickness of 30 μm, then stored at -20 °C until needed for staining. The sectioning plane of mouse temporal bones was oriented such that the cutting face was parallel to the AN within the cochlear modiolus to ensure that sections were mid-modiolar to allow optimal viewing of the AN GTZ.

### 2.4 Human cochlear tissue collection and preparation

Procedures for the collection and preparation of human temporal bones have been previously reported (39–41). All specimens were obtained from the MUSC Hearing Research Program’s temporal bone archive and the MUSC Carroll A. Campbell, Jr. Neuropathology Laboratory Brain Bank. In all cases of human temporal bone collection, written informed consent was obtained from the next-of-kin in accordance with South Carolina laws and regulations. Temporal bone research was approved by the MUSC Institutional Review Board as not human subject research (Pro0030845). No hearing history is available for the donors used in this study. **Table 1** lists the age, sex, and time interval between death and fixation of the human temporal bones in this study. After removal of the temporal bone from the skull, scalar perfusion was performed with a 4% solution of paraformaldehyde. Fixation was continued by immersion for at least 48 hours at 4 °C. Each temporal bone was then rinsed with 1x PBS and decalcified in 0.35 M EDTA, pH 8.0 for a period of 4–6 weeks as described previously (39). During this decalcification period, each specimen was trimmed to remove the hard bone encasing the cochlea and vestibule of the inner ear. Once fully decalcified and trimmed, the inner ear portions of the temporal bone were processed for frozen sectioning at a thickness of 12 μm.

**Table 1:** Temporal bone donor information.

ID	Age	Sex	Postmortem fixation interval (hours)
H85	59	Male	16
H55	86	Male	4.8
H51	89+	Female	>20

### 2.5 Immunohistochemistry on mouse and human cochlear sections

Cochlear sections were air dried with a fan for 30 minutes, then submerged in -20 °C acetone for 5 minutes followed by -20 °C methanol for 10 minutes. Sections were permeabilized and blocked in donkey serum buffer with Triton X-100 (16% donkey serum, 0.3% Triton X-100, 450 mM NaCl, 20 mM Phosphate Buffer) at room temperature for 3 hours. Primary antibodies were prepared in donkey serum buffer with Triton X-100 and added to the sections, then incubated overnight at 4 °C. The next day, sections were washed with wash buffer with Triton X-100 (0.3% Triton X-100, 450 mM NaCl, 20 mM Phosphate Buffer), and the appropriate biotinylated secondary antibodies conjugated with fluorescent avidin (Vector Laboratories) were added as previously described (42). For myelin staining, slides were washed, blocked, and then stained with FluoroMyelin™ (ThermoFisher Scientific) overnight at 4 °C, based on the protocol recommended by the manufacturer. The following day, slides were washed using wash buffer with Triton X-100, stained for nuclei (Hoechst), then washed again before being mounted with Vectashield (Vector Laboratories). The primary antibodies, secondary antibodies, and stains used for immunohistochemistry are listed in **Table 2**.

**Table 2:** Antibodies and stains.

Antibody	Host	Vendor	Catalog number	Dilution
Alexa Fluor™ 488 anti-mouse IgG	Goat	ThermoFisher Scientific	A11029	1:200
Alexa Fluor™ 647 anti-mouse IgG	Donkey	ThermoFisher Scientific	A31571	1:200
Alexa Fluor™ 647 anti-rat IgG	Donkey	ThermoFisher Scientific	A78947	1:100
Biotinylated anti-goat IgG	Rabbit	Vector Laboratories	BA-5000	1:100
Biotinylated anti-rabbit IgG	Goat	Vector Laboratories	BA-1000	1:100
Biotinylated anti-rat IgG	Rabbit	Vector Laboratories	BA-4000	1:100
CD107a (Lamp1)	Rat	ThermoFisher Scientific	14-1071-85	1:100
CD163	Rat	BioLegend	155302	1:100
CD206, Alexa Fluor® 647	Rat	BioRad	MCA2235A647	1:100
CD68, REAfinity Vio G570	N/A	Miltenyi Biotec	130-133-803	1:50
FluoroMyelin™ Green	N/A	ThermoFisher Scientific	F34651	1:700
Galectin-3 (Gal3)	Goat	R&D Systems	AF1197	1:100
Glial Fibrillary Acidic Protein (GFAP)	Mouse	Millipore	MAB360	1:200
Hoechst 33342	N/A	ThermoFisher Scientific	H3570	1:500
Iba1	Rabbit	Abcam	AB178847	1:150
Myelin Basic Protein (MBP), REAfinity Vio R667	N/A	Miltenyi Biotec	130-131-152	1:50
NG2	Rabbit	Millipore	AB5320	1:100
Streptavidin Fluorescein	N/A	Vector Laboratories	SA5001	1:100
Streptavidin Texas Red™	N/A	Vector Laboratories	SA5006	1:100
Toluidine Blue	N/A	Millipore	89640	1%

### 2.6 Confocal imaging with Airyscan and post-acquisition analysis

Slice and confocal image stacks were collected on a Zeiss LSM 880 NLO with Airyscan using ZEN acquisition software (Zeiss United States) or an Andor BC43 Spinning Disk Confocal using Fusion Benchtop acquisition software (Oxford Instruments). For low magnification human cochlear section preparations, images were taken with a 20x air objective at a resolution of 0.52 µm x 0.52 µm to evaluate the AN and AN GTZ. High magnification human AN images were taken with a 63x oil objective at a resolution of 0.16 µm x 0.16 µm x 0.60 µm. Imaging was performed at a resolution of 0.16 µm x 0.16 µm to analyze macrophage/microglia morphology and myelination in human temporal bone sections. For mouse cochlear section preparations, images were taken at a resolution of 0.83 µm x 0.83 µm for evaluating the AN and AN GTZ. For analysis of macrophage/microglia morphology and myelination (including assessment of internalized myelin), image stacks were taken at a resolution of 0.13 µm x 0.13 µm with a z-step of 0.60 µm. Images were processed using ZEN 3.9 Blue Edition (Carl Zeiss Microscopy GmbH).

The Imaris volume rendering function (Imaris 10.2, Oxford Instruments) was used for 3D reconstructions and analysis of macrophage/microglia morphology, myelination, and identification and quantification of internalized myelin within AN macrophages/microglia.

Immunofluorescence image stacks from 30 µm sections in CBA/CaJ mice of both age groups were imported into Imaris 10.2. For the 3D reconstruction of Iba1^+^ cells, surfaces were created using the Machine Learning Segmentation Tool (Imaris 10.2, Oxford Instruments). Training was performed on each image using 3–5 repetitions as needed, based on complexity of macrophage/microglia morphology. Any touching objects were split. Surfaces were hand-filtered to remove any objects that were not part of the Iba1^+^ macrophage/microglia mask. All surface parameters were exported into a separate .csv file for each image stack. Surface area and volume of Iba1^+^ cells per 1 mm^2^ were determined for young (n = 4) and aged (n = 4) mice. Average number of Iba1^+^ cells per region of interest (ROI; 131.79 µm x 131.79 µm) and average number of nuclei per ROI (65.90 µm x 65.90 µm) were also calculated for young (n = 3) and aged (n = 3) mice.

Myelination in young (n = 4) and aged (n = 4) mice was quantified in the same manner, using the Machine Learning Segmentation Tool (Imaris 10.2, Oxford Instruments), which generated surfaces of each myelinated fiber in the section and output the surface area and volume of the myelinated fibers contained within each ROI (131.79 µm x 131.79 µm). For the 3D reconstruction of myelinated fibers in the AN to evaluate surface area and volume, the same procedure was used as described above for generating surfaces of Iba1^+^ macrophages/microglia. After the surfaces of the myelinated fibers were created for each image, the statistics were exported, including the total volume and surface area of each surface. All surface parameters were exported into a separate .csv file for each image stack.

The quantity of internalized myelin was determined based on the volume of the myelin surface contained within the Iba1^+^ surface to ensure only fully enclosed myelin components were included in the analysis. For quantification of total internalized myelin within Iba1^+^ macrophages/microglia, Object-Object Statistics were performed in Imaris (Imaris 10.2, Oxford Instruments). For each individual image, the Iba1^+^ macrophage/microglia surface was used to create a mask against the myelinated fiber surface generated for each stack. Then, a surface was created that contained only myelin-stained objects within the Iba1^+^ macrophage/microglia surface. The statistics for the internalized myelin surface were then exported to determine total volume of internalized myelin within the Iba1^+^ macrophages/microglia for each image stack.

### 2.7 Epoxy resin embedding preparation of the AN

CBA/CaJ mice were processed as previously described (42). Briefly, mice underwent cardiac perfusion with 10 mL normal saline with 0.1% sodium nitrate, then 15 mL of 4% paraformaldehyde and 2% glutaraldehyde in 0.1 M phosphate buffer, pH 7.4. Cochleae were decalcified in 50 mL of 120 mM solution of EDTA, pH 7.2 with gentle stirring at room temperature for 2–3 days and daily changes of EDTA solution. Tissues were post-fixed in 1% osmium tetroxide for 1 hour, dehydrated, and embedded in Epon LX 112 resin. Semi-thin sections were cut at approximately 1 µm thick and stained with Toluidine Blue.

### 2.8 Quantification of the AN fibers

Transversely sectioned images of the AN were used for quantification of the AN fiber density, axon diameter, myelinated fiber (axon + myelin) diameter, myelin sheath thickness, and g-ratio. All images for these analyses were taken using an Olympus BX53 with a 60x oil objective. To calculate the density of AN fibers, this study used 4 young and 4 aged CBA/CaJ mice. Eight ROIs were selected for each mouse including 4 peripheral and 4 central ROIs to ensure equal representation of the central and peripheral AN. Fiber density was then calculated as quantity of fibers per 1 mm^2^.

For measures of fiber size and g-ratio, young (n = 4) and aged (n = 4) CBA/CaJ mice were used with 8 ROIs per animal, with equal numbers of central and peripheral ROIs. Each ROI included at least 100 fibers randomly selected throughout the ROI. Fibers were only measured if the perimeter of the axon and the perimeter of the myelin sheath were clearly distinguishable. Diameters of the myelinated fiber (axon + myelin) and the axon were measured manually for each fiber using FIJI ImageJ. Myelin sheath thickness was calculated as half the difference between the axon and myelinated fiber diameter. G-ratio was calculated as the ratio of the axon diameter divided by myelinated fiber diameter (43, 44).

### 2.9 Light sheet microscopy and three-dimensional high-resolution imaging of the AN GTZ

Young (n = 3) and aged (n = 3) mouse temporal bones were tissue-cleared and imaged with large-field light sheet microscopy to visualize the AN GTZ. Tissue clearing was performed using the MACS Clearing Kit (Miltenyi Biotec) and their standard protocol with the addition of a decalcification step (10% EDTA for 1 day) before permeabilization. Mouse temporal bones were stained with CD68 (Miltenyi Biotec) and Myelin Basic Protein (MBP) (Miltenyi Biotec). Detailed descriptions of the antibodies used are listed in **Table 2**. Cleared whole mouse temporal bones were mounted and imaged with an Ultramicroscopy II Scope (Miltenyi Biotec) with a Super Plan configuration, equipped with a sCMOS camera (4.2 Megapixel, Andor Technology), and objective lenses with dipping caps specifically designed and optimized for large-field light sheet imaging as previously described (45). All 3D image stacks were acquired using a 561 nm laser with a 595/40 nm emission filter to capture CD68 fluorescence, and a 639 nm laser with a 680/30 nm emission filter to capture MBP fluorescence. Each mouse temporal bone was mounted on the sample holder and oriented such that volume was acquired with the z-steps moving from apex to base through the AN to ensure the capture of the full AN GTZ. The entire AN GTZ was imaged using a 12x objective at 0.6x magnification with a planar pixel size of 0.903 µm x 0.903 µm (X,Y) with a 1 µm z-step interval. The high-resolution 3D imaging of the AN GTZ was performed using the same 12x objective at 1.66x magnification, also with a 1 µm z-step interval. CD68^+^ cells were quantified within the AN for each temporal bone from the light sheet volumes in young and aged CBA/CaJ mice.

### 2.10 RNA-sequencing analysis of the AN in young and aged mice

To examine expression of genes relating to myelination, glial cell function, and abnormal inflammation in young and aged mouse AN, analysis was performed on bulk RNA-sequencing data obtained from a prior study involving young adult (3 months; n = 3) and aged (>2.5 years; n = 3) ANs from CBA/CaJ mice (accession GSE141865) (34). AN tissue in the previous study were isolated by microdissection of the modiolus from the other structures of the cochlear bulla (such as the cochlear lateral wall and sensory epithelium), with the tissue collected from the two cochleae of each mouse pooled to make each biological replicate; three biological replicates of each age group were used. Raw sequencing data (fastq files) were analyzed using Partek Flow software (Illumina Inc). Reads were aligned to the mouse genome assembly mm39 by STAR (version 2.7.8a) (46) and quantified to annotation model (Partek E/M) built from mm39 Ensembl Transcripts release 104 using default parameter settings (Strict paired-end compatibility=true; Require junction reads to match introns=true; Minimum read overlap with feature=100%). Normalization and comparative analysis was done with DESeq2 (47). Normalized count data is archived in NCBI Gene Expression Omibus (accession GSE320402). Differential gene expression was defined as adjusted *p*-value (FDR step up) < 0.05 and absolute fold change > 2, yielding 1240 genes (**Supplementary Table 1**). Biological process enrichment analysis was conducted with ToppGene (48).

### 2.11 Statistical analysis

Sample sizes for experiments and morphological observations are listed in the appropriate results section and figure legends. Images presented here are representative of each experimental group for fluorescence staining and Toluidine Blue sections. All statistical values are presented as a mean ± standard deviation unless stated otherwise. All data sets were tested for normality using a Kolmogorov-Smirnov test with reference to a normal distribution. Mann-Whitney U-test was used for pairwise comparisons between groups. Exact *p*-values are reported for all comparisons. For differential expression analyses of RNA-sequencing data, a *p*-adjusted value (FDR step up) of < 0.05 and absolute fold change > 2 was considered significant. Graphs were plotted and statistics were performed in Igor Pro 9 (Wavemetrics) and GraphPad Prism 8 (GraphPad Software).

## 3 Results

Previous studies on aging effects on neural degeneration of the auditory system have focused on the peripheral AN fibers in the region where they project to the sensory epithelia, specifically on the spiral ganglion and ribbon synapses within the inner hair cells (5, 34, 49–52). Other studies have focused on the central auditory system, including the cochlear nucleus and auditory cortex (24, 53–55). Here, this study investigated aging effects on AN myelination and inflammation, focusing on the GTZ which houses both peripheral and central immune cells and myelinating glia, in an established ARHL mouse model and in human temporal bone sections from three older donors (aged 59, 86, and 89+ years).

### 3.1 The glial transition zone of the mouse AN

**Figure 1:**
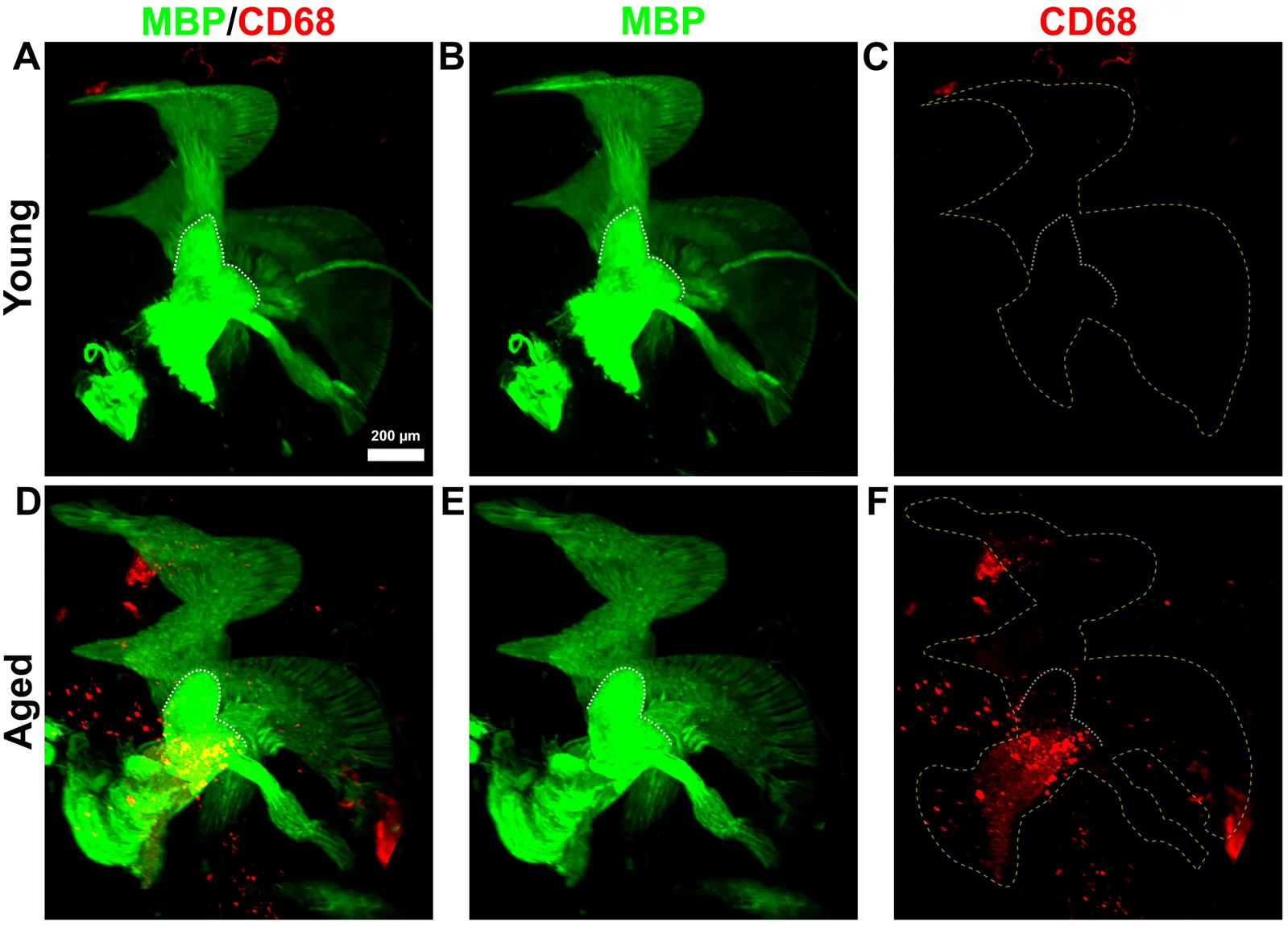
Large-field light sheet microscopy reveals the central-peripheral interface within the AN GTZ. **(A–F)** Three-dimensional renderings of cleared whole mouse temporal bones from young **(A–C)** and aged **(D–F)** CBA/CaJ mice. Temporal bones were stained with MBP [green; **(A, B, D, E)**] and macrophage/microglia activation marker CD68 [red; **(C, F)**]. The AN GTZ is indicated by a white dashed line.

To evaluate the GTZ of the AN and its immune landscape in its entirety, we utilized large-field light sheet microscopy of whole mouse temporal bones of young and aged CBA/CaJ mice (**Figure 1**). Temporal bones were immunostained with a fluorescently tagged MBP antibody, a reliable marker of AN myelin sheaths (40). Examining young and aged mouse temporal bones showed that the GTZ spans the entirety of the AN and resides within the cochlear modiolus in a cone-shaped morphology (**Figures 1A, B, D, E**). A visible transition from peripheral AN to central AN is evident based on the reduced MBP staining intensity in the peripheral AN compared to the central AN in both the young and aged samples. Increased central myelin staining by MBP is consistent with known compositional differences between central and peripheral myelin. Previous studies report that MBP makes up approximately 30% of the protein mass of central myelin, while it only accounts for 5–18% of the protein mass of peripheral myelin (28, 56). To identify activated immune cell subpopulations with phagocytic activity, temporal bones were also stained with a fluorescently tagged CD68 antibody (57–59). CD68 is an established marker of phagocytic activity and immune activation for both macrophages and microglia (60). More CD68^+^ cells were observed within the AN in the aged temporal bones compared to the young temporal bones, mostly within the central AN and aggregated near the AN GTZ in the basal turns (**Figures 1C–F**).

**Figure 2:**
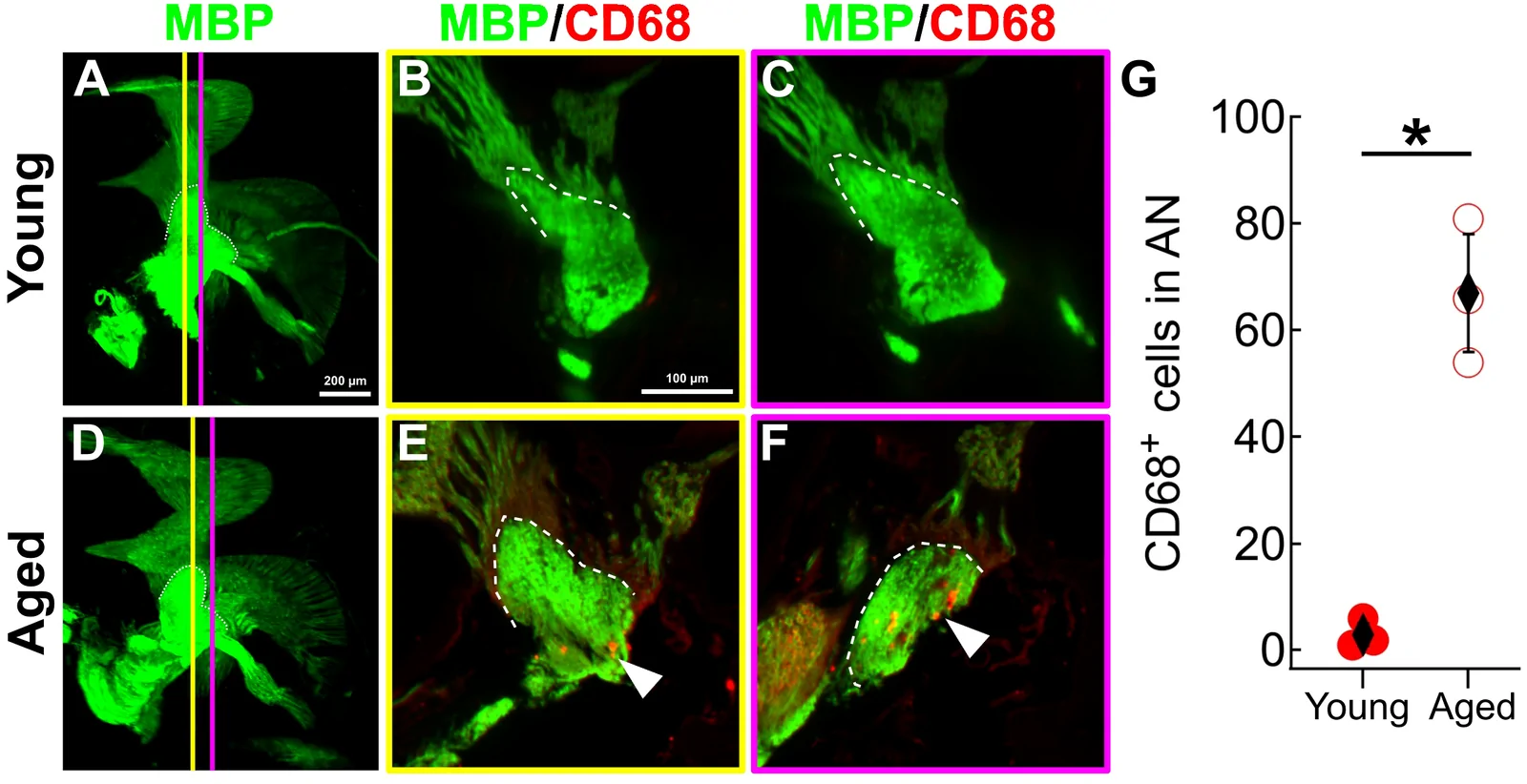
Light sheet datasets enable analysis of the whole AN and reveal age-related accumulation of CD68^+^ cells in the AN. **(A–F)** Serial sections from cleared mouse temporal bones imaged by large-field light sheet microscopy show the entire AN within the cochlea and the AN GTZ in young **(A–C)** and aged **(D–F)** CBA/CaJ mice. Section locations are indicated by the yellow and magenta lines in the corresponding three-dimensional renderings in **(A, D)**. Each temporal bone was stained with MBP (green) and CD68 (red). White dashed lines indicate the cone-shaped morphology of the AN GTZ. In F, the white dashed line marks the GTZ in the basal turn/hook region of the cochlea. White arrows in **(E, F)** indicate increased CD68^+^ cells within the aged AN, whereas few CD68^+^ cells were observed in the young AN. **(G)** Quantification of CD68^+^ cells throughout the entire AN in young (n=3) and aged (n=3) mice. All distributions were assessed for normality using a Kolmogorov-Smirnoff test. Statistical significance was determined using a Mann-Whitney U test, α = 0.05. Diamonds indicate the mean, and error bars represent standard deviation. *p < 0.05.

Large-field light sheet microscopy of tissue cleared mouse temporal bones was used to quantify CD68^+^ cells within the entire intact AN and AN GTZ. Whole mouse temporal bone light sheet datasets allow complete visualization of the AN GTZ and all CD68^+^ cells within the AN (**Figures 2A–F**; **Supplementary Figure 2**). Aged mice had a significantly greater number of CD68^+^ cells within the AN compared to young mice (AVG_Young_ = 3.00 ± 2.16, AVG_Aged_ = 67.00 ± 11.05, *p* = 0.025) (**Figure 2G**).

**Figure 3:**
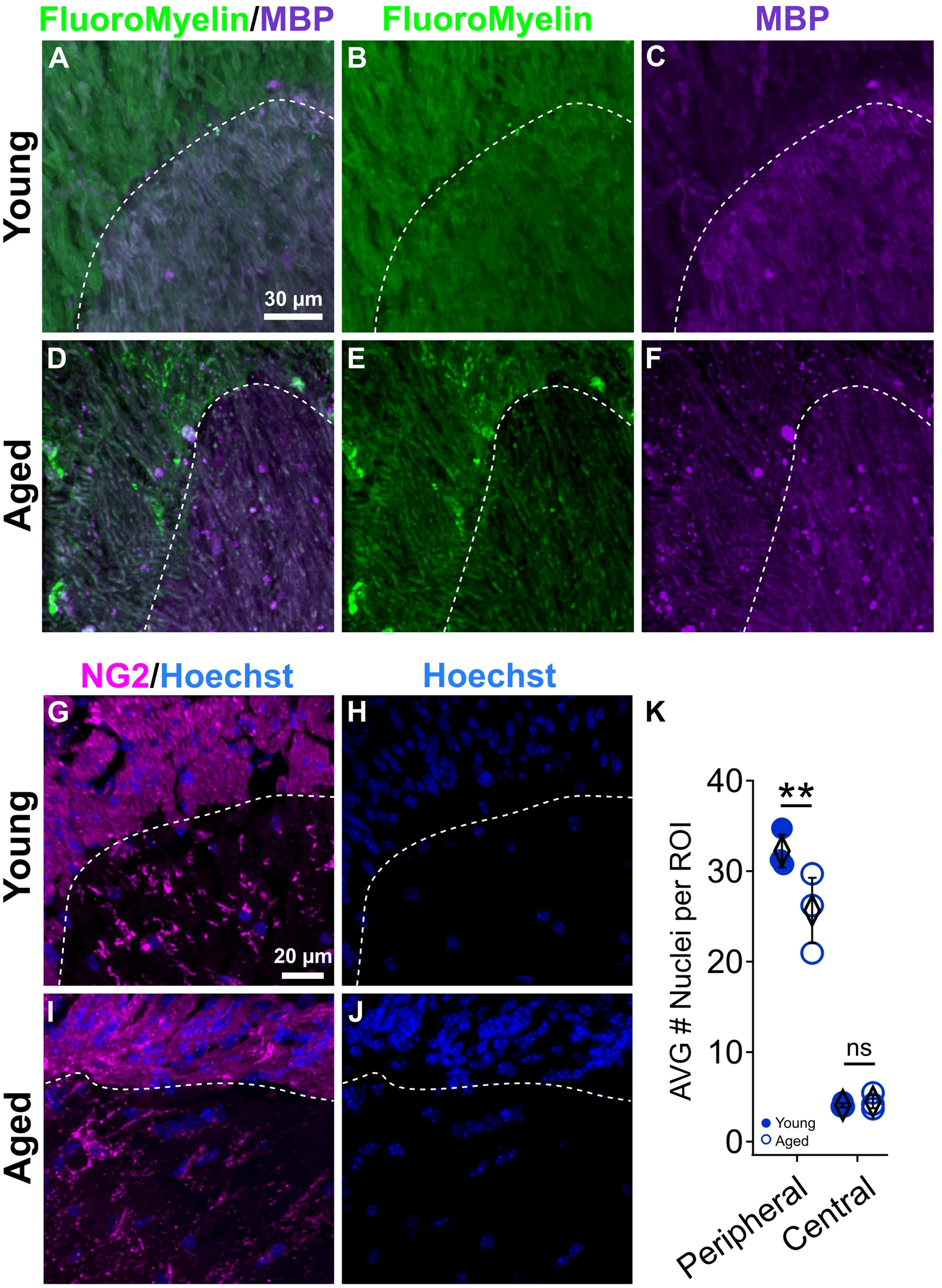
The AN GTZ delineates the interface between central and peripheral myelination within the cochlea. **(A–F)** Thirty-micron mid-modiolar sections of the AN GTZ from young and aged CBA/CaJ mice. Sections are stained with FluoroMyelin™ (green) and MBP (violet). **(G–J)** Thirty-micron mid-modiolar sections of the AN GTZ from young and aged CBA/CaJ mice stained for the oligodendrocyte progenitor cell marker NG2 (magenta) and Hoechst nuclear stain (blue). The white dashed line indicates the location of the AN GTZ. **(K)** Average number of nuclei per region of interest (ROI) in the peripheral and central AN in young (filled blue circles) and aged (open blue circles) mice. N = 3 mice per group with 4 ROIs analyzed per mouse. All distributions were evaluated for normality using a Kolmogorov-Smirnoff test. Statistical significance was determined using a Mann-Whitney U test, α = 0.05. Open diamonds show the mean, and error bars represent standard deviation. **p < 0.01.

The AN GTZ is a true central-peripheral boundary where myelination transitions from Schwann cells in the peripheral AN to oligodendrocytes in the central AN. Immunostaining of 30 µm sections with FluoroMyelin™ and MBP revealed the GTZ boundary and showed concordant labeling of myelin structures (**Figures 3A–F**). The peripheral AN showed stronger FluoroMyelin™ stain and the central AN demonstrated stronger MBP stain, which was expected based on differences in peripheral and central myelin composition, confirming the observation revealed in light sheet imaging (**Figure 1**). To further confirm central vs peripheral myelination, sections were also stained for NG2, an oligodendrocyte progenitor cell marker (61), which labeled cell somas on the central side of the AN GTZ (**Figures 3G–J**). Interestingly, NG2 immunoreactivity was also observed more diffusely within myelin sheaths in the peripheral AN compared to the central NG2^+^ somas, consistent with previous studies demonstrating NG2 expression in cells of Schwann cell lineage under certain conditions (62, 63).

The distinction between peripheral and central is further validated by the differences in nuclear density between the peripheral and central AN (**Figure 3K**). We quantified nuclear density within the peripheral and central regions of the AN of young and aged mice. Like other cranial nerve transition zones, the higher nuclear density seen in the peripheral AN compared to the central AN reflects the differences in myelinating cell types across the GTZ (64). Young mice had a significantly greater nuclear density in the peripheral AN compared to aged mice (Peripheral AN: AVG_Young_ = 32.35 ± 1.78, AVG_Aged_ = 25.67 ± 3.60, *p* = 0.0024). There was no significant difference in nuclear density in the central AN between young and aged mice (Central AN: AVG_Young_ = 4.17 ± 0.24, AVG_Aged_ = 4.50 ± 0.74, *p* = 0.59).

### 3.2 Aging ANs have more immune cells with focal accumulation at the GTZ

**Figure 4:**
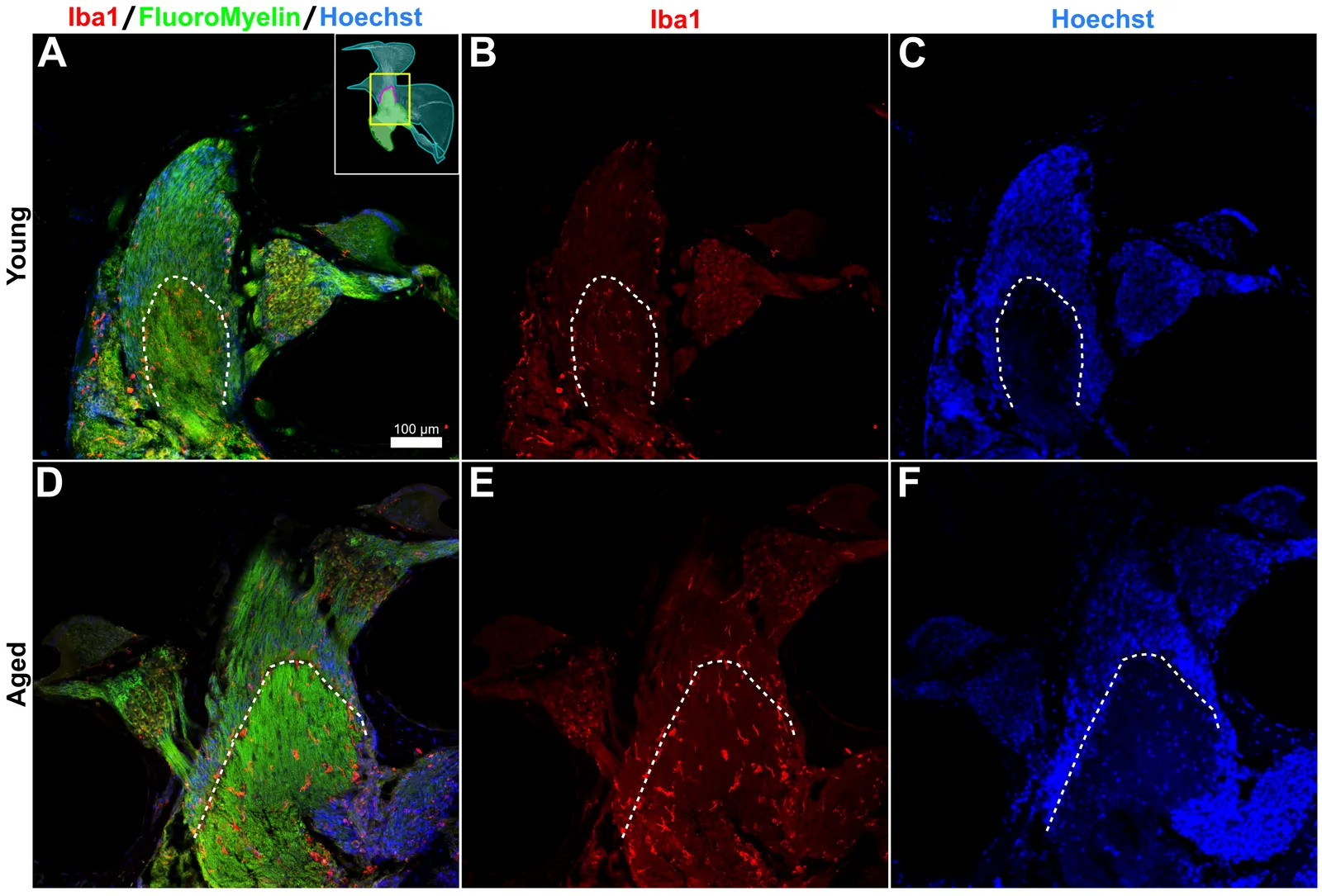
Mouse mid-modiolar sections reveal age-related changes to the AN GTZ. **(A–F)** Thirty-micron mid-modiolar sections of AN GTZ in a young **(A–C)** and aged **(D–F)** CBA/CaJ mice. Sections were stained with FluoroMyelin™ (green), macrophage/microglia cell marker Iba1 (red), and Hoechst nuclear stain (blue). The white dashed line indicates the location of the AN GTZ in mid-modiolar section. Scale bar = 100 µm.

To better evaluate the age-related changes of AN macrophages/microglia and myelination of the AN GTZ, we utilized high-resolution imaging of FluoroMyelin™-stained 30 µm frozen sections of young and aged CBA/CaJ mice (**Figures 4A–F**; **Table 2**). Sections were stained with Ibal, an established marker of macrophages and microglia, and a nuclear marker (Hoechst) to aid in the identification of the cell bodies of Iba1^+^ macrophages/microglia. Iba1 staining showed macrophages and microglia dispersed throughout the AN in young and aged sections (**Figures 4B, E**). In these low-magnification mid-modiolar sections, observable differences in the Iba1^+^ cell populations throughout the AN and at the GTZ are apparent, with the aged sections having a greater number of Iba1^+^ cells throughout the AN and appearing to accumulate at the GTZ (**Figures 4B, E**).

**Figure 5:**
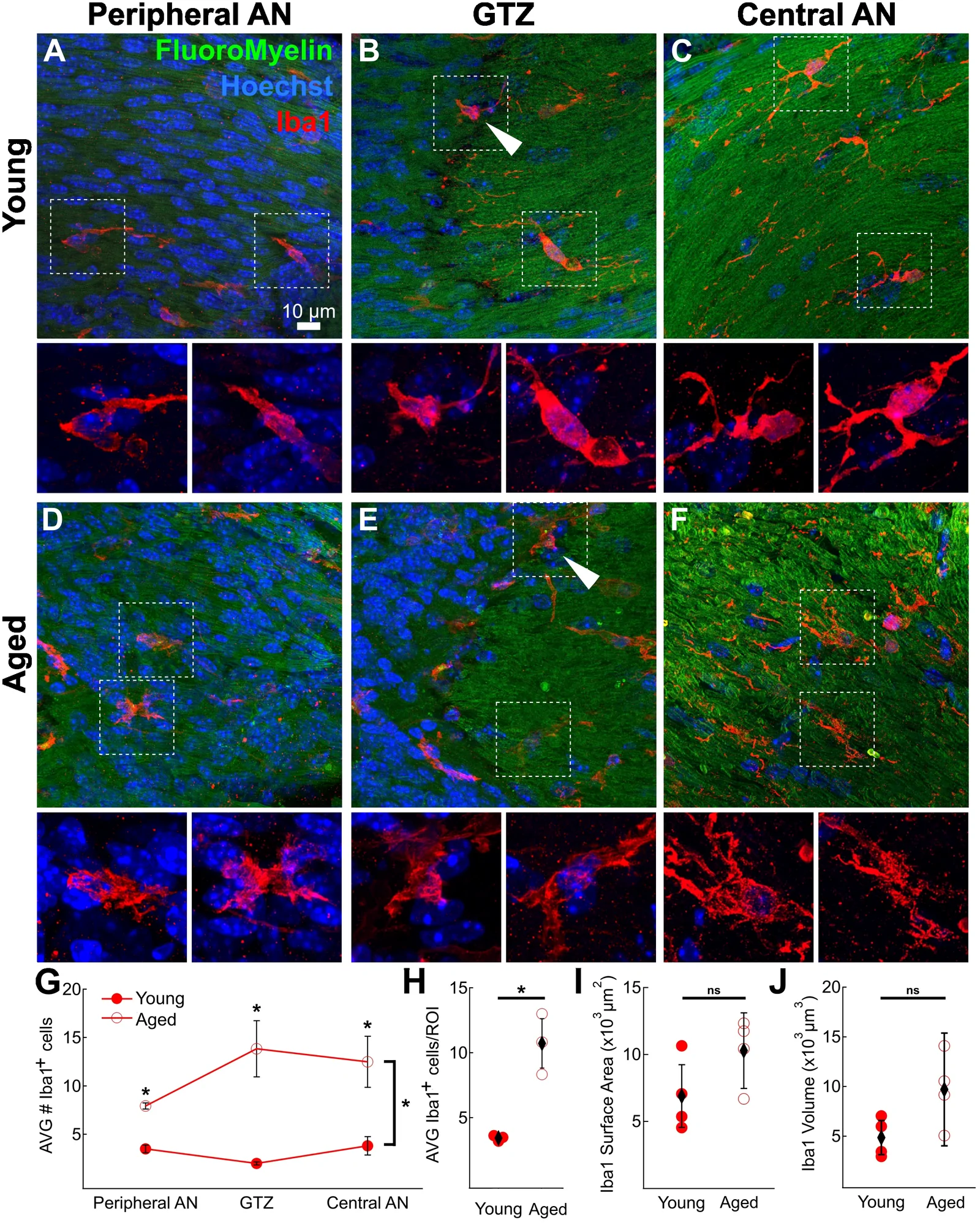
Increased density of immune cells at the AN GTZ in aged mice. **(A–F)** Immunostained sections of the AN in young **(A–C)** and aged **(D–F)** mice showing Iba1^+^ cells in the peripheral AN **(A, D)**, AN GTZ **(B, E)**, and central AN **(C, F)**. White arrows indicate Iba1^+^ cells that span the peripheral and central regions of the AN across the GTZ. **(G)** Average number of Iba1^+^ cells in the peripheral AN, AN GTZ, and central AN in young and aged mice. N = 3 mice per group, with 3 ROIs analyzed per region per age group. **(H)** Average number of Iba1^+^ cells per ROI in young and aged mice. N = 3 per group with 9 ROIs analyzed per mouse (3 ROIs in the peripheral AN, 3 ROIs in the central AN, and 3 ROIs in the AN GTZ). **(I)** Average surface area of Iba1^+^ cells per ROI in young (filled red circles) and aged (open red circles) mice. N = 4 mice per group, with 4 ROIs analyzed per mouse (1 ROI in the peripheral AN, 1 ROI in the central AN, and 2 ROIs in the AN GTZ). **(J)** Average volume of Iba1^+^ cells per ROI in young and aged mice. N = 4 mice per group, with 4 ROIs analyzed per mouse (1 ROI in the peripheral AN, 1 ROI in the central AN, and 2 ROIs in the AN GTZ. All distributions were evaluated for normality using a Kolmogorov-Smirnoff test. Significance was determined using a Mann-Whitney U test, α = 0.05. Diamonds show the mean, and error bars represent standard deviation. *p < 0.05.

Immunostained sections of the peripheral AN, AN GTZ, and central AN are shown in young (**Figures 5A–C**) and aged (**Figures 5D–F**) mice. Of all three regions of the AN, the GTZ had the greatest density of Iba1^+^ cells in aged mice compared to young mice (Peripheral AN: AVG_Young_ = 3.50 ± 0.41, AVG_Aged_ = 7.93 ± 0.32, *p* = 0.0247; GTZ: AVG_Young_ = 2.00 ± 0.16, AVG_Aged_ = 13.85 ± 2.90, *p* = 0.0463; Central AN: AVG_Young_ = 3.83 ± 0.94, AVG_Aged_ = 12.52 ± 2.64, *p* = 0.0463) (**Figure 5G**). Aged mice contained significantly more Iba1^+^ cells compared to young mice across all regions of the AN (AVG_Young_ = 3.44 ± 0.18, AVG_Aged_ = 10.72 ± 1.91, *p* = 0.0317) (**Figure 5H**). Interestingly, Iba1^+^ cells in the AN GTZ were also found with projections across both central and peripheral regions of the AN (**Figures 5B, E**).

To evaluate the activation state of Iba1^+^ cells within the AN, given that larger size can be a sign of increased activation, we assessed their morphology, including surface area and volume (65, 66). There were no significant differences in Iba1^+^ cell surface area or volume between age groups. (Surface Area: AVG_Young_ = 6.90 ± 2.35 x 10^3^ µm^2^, AVG_Aged_ = 10.29 ± 2.83 x 10^3^ µm^2^, *p* = 0.111; Volume: AVG_Young_ = 4.86 ± 1.71 x 10^3^ µm^3^, AVG_Aged_ = 9.71 ± 5.67 x 10^3^ µm^3^, *p* = 0.064) (**Figures 5I, J**).

**Figure 6:**
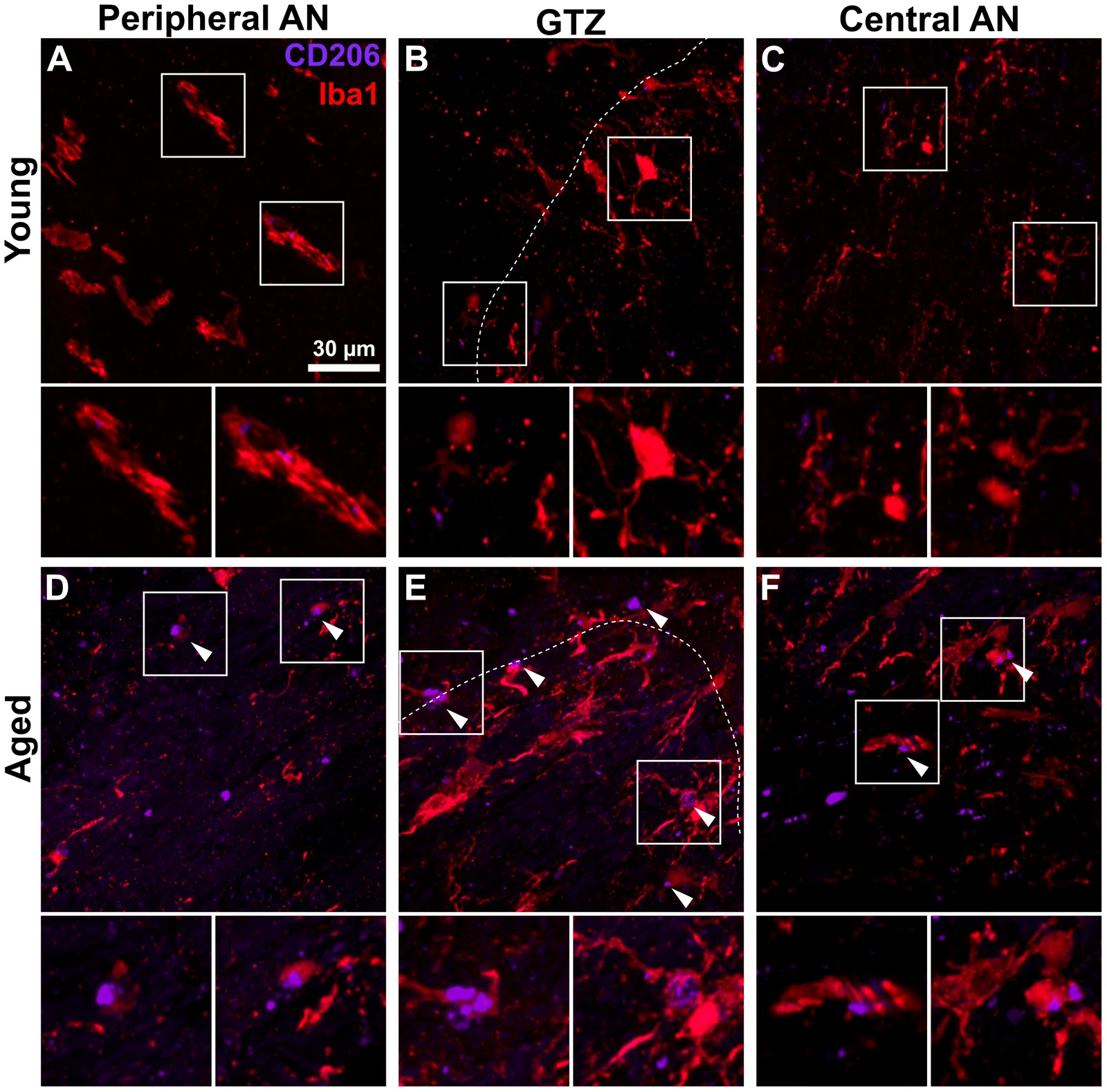
Aging is associated with increased CD206^+^ Iba1^+^ cells and their accumulation at the AN GTZ. **(A–F)** Immunostained sections of the AN in young **(A–C)** and aged **(D–F)** mice showing CD206^+^ Iba1^+^ cells in the peripheral AN **(A, D)**, AN GTZ **(B, E)**, and the central AN **(C, F)**. White arrows indicate cells that are CD206^+^ Iba1^+^ in the AN, which are more abundant in aged mice and are predominantly observed at the AN GTZ. White dashed lines indicate the location of the GTZ with the peripheral AN at the top of each image.

To further evaluate age-related changes in the Iba1^+^ population within the AN, we stained for the mannose receptor 1, CD206, which is involved with endocytic processes and important to detection of glycoproteins and polysaccharide chains (67, 68). CD206 is an established marker of macrophage activation with increased expression in the aging brain (69). We observed more CD206^+^ cells within the AN in aged mice compared to young mice (**Figures 6A–F**). Additionally, when comparing the peripheral AN, GTZ, and central AN, more CD206^+^Iba1^+^ cells with larger CD206 inclusions were observed with aging at the AN GTZ (**Figures 6B, E**). We evaluated additional markers of immune activation, including CD163 and Gal3, and observed age-related increases in both markers, with CD163 found only at the AN GTZ (32, 70) (**Supplementary Figures 3**, **4**).

### 3.3 Aging is associated with decreased AN fiber density and disruption of AN myelin

**Figure 7:**
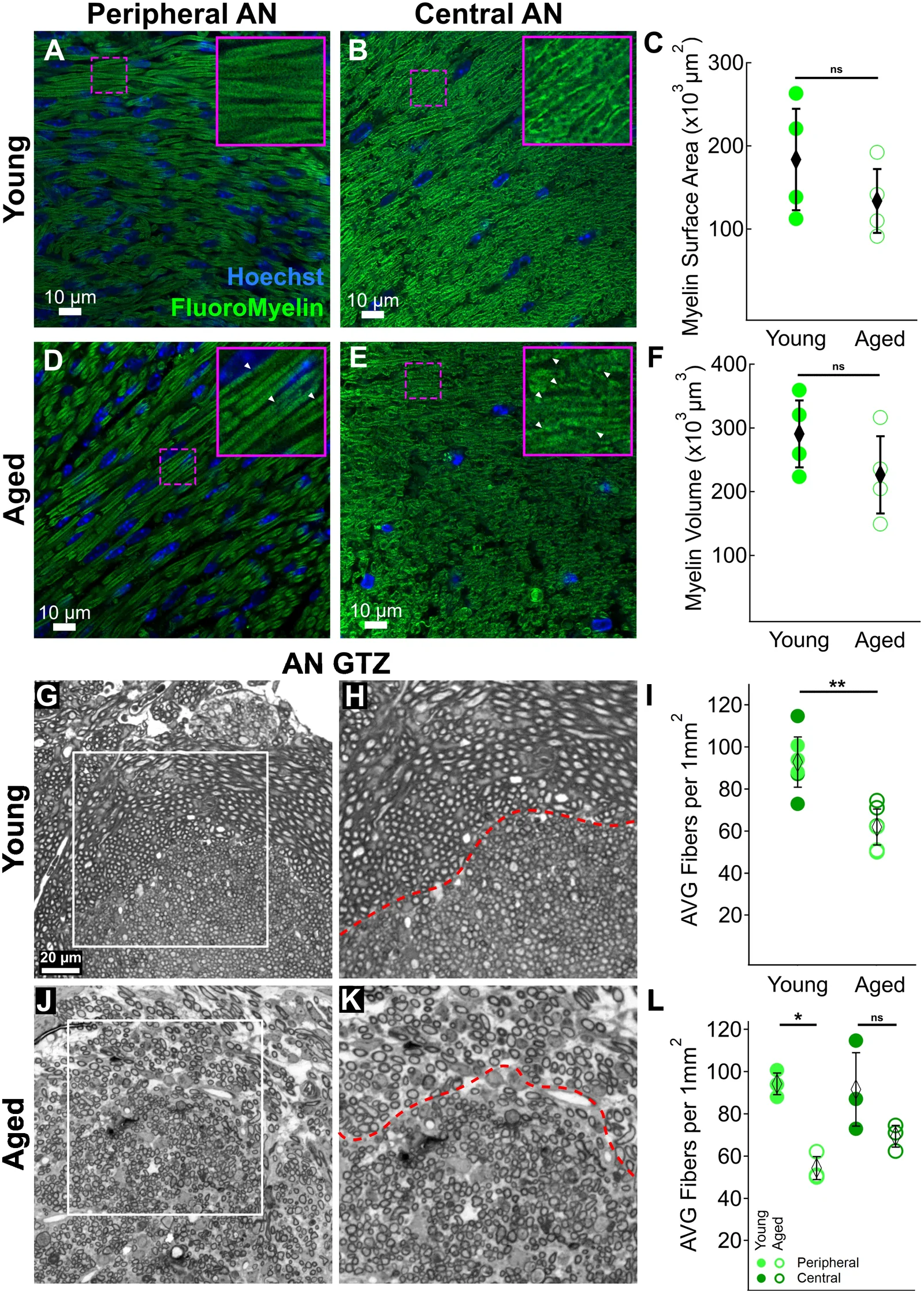
Aging is associated with disrupted myelin sheath morphology and reduced AN fiber density. **(A, B)** FluoroMyelin™-stained sections in the peripheral AN **(A)** and central AN **(B)** from young and aged CBA/CaJ mice. Insets in the upper right show enlarged views of the dashed boxed regions in **(A–C)** Quantification of myelinated AN fiber surface area in young (filled green circles) and aged (open green circles) mice. N = 4 mice per age group, with 4 ROIs per mouse (2 ROIs in the peripheral AN, 2 ROIs in the central AN). The difference between the young and the aged mice was not significant. **(D, E)** FluoroMyelin™-stained sections of the peripheral AN **(D)** and central AN **(E)** from aged CBA/CaJ mice. Insets in the upper right show enlarged views of the dashed boxed regions in **(D, E)** White arrows indicate the disrupted myelin sheaths in the peripheral AN **(D)** and central AN **(E, F)** Quantification of myelinated AN fiber volume in young and aged mice. N = 4 mice per age group, 4 ROIs analyzed per mouse. The difference between the young and the aged was not significant. **(G–L)** Epoxy resin-embedded sections stained with Toluidine Blue showing the AN GTZ in a young **(G, H)** and aged **(J, K)** mouse. Red dashed lines indicate the AN GTZ, with the peripheral AN oriented toward the top of the images. White boxes in **(G, J)** indicate the enlarged regions shown in H and K, respectively. Quantification of average AN fiber density per 1 mm^2^ in young and aged mice **(I)**. Quantification of the average AN fiber density per 1 mm^2^ in the peripheral and central regions of the AN in young and aged mice **(L)**. N = 3 mice per group, 8 ROIs per animal (equal number of central and peripheral ROIs). All distributions were evaluated for normality using a Kolmogorov-Smirnoff test. Significance was determined using a Mann-Whitney U test, α = 0.05. Diamonds show the mean, and error bars represent standard deviation. *p < 0.05. **p < 0.01. Scale bar in **(A, B, D, E)** = 10 µm.

Myelin staining revealed differences in morphology between the peripheral AN and central AN in young and aged mice (**Figures 1**-**5**). The peripheral AN had a homogenous myelin stain, while the central AN had a more heterogeneous appearance in both young and aged mice (**Figure 7**). We observed more disrupted myelin sheaths throughout the peripheral and central AN regions with age indicated by the unilateral gaps in the myelin sheath (**Figures 7A, B, D, E**), which confirms previous electron microscopy findings in the peripheral AN (34, 40). To quantify age-related changes in myelination in the AN, we measured the volume and surface area of myelinated fibers in young and aged CBA/CaJ mice using Imaris. Aged mice had no significant difference in surface area or volume of myelinated fibers compared to young mice (**Figures 7C, F**) (Surface Area: AVG_Young_ = 183.75 ± 61.00 x 10^2^ µm^2^, AVG_Aged_ = 133.74 ± 38.30 x 10^2^ µm^2^, *p* = 0.342; Volume: AVG_Young_ = 290.74 ± 52.63 x 10^3^ µm^2^, AVG_Aged_ = 226.50 ± 60.45 x 10^3^ µm^2^, *p* = 0.200).

To further assess age-related changes in AN degeneration, we calculated the density of AN fibers in the peripheral AN and central AN in young and aged CBA/CaJ mice (**Figures 7G–L**). Sections of the AN GTZ stained with Toluidine Blue in young and aged mice show the difference in AN fiber density in the peripheral and central regions of the AN with aging (**Figures 7G, H, J, K**). Young mice had a significantly greater AN fiber density (including both the peripheral and central regions of the AN) compared to aged mice (AVG_Young_ = 92.96 ± 11.93 fibers/1 mm^2^, AVG_Aged_ = 61.93 ± 8.51 fibers/1 mm^2^, *p* = 6.49 x 10^-3^) (**Figure 7I**). Young mice had significantly greater peripheral AN fiber density compared to aged, while the central AN fiber density had no significant difference (Peripheral AN: AVG_Young_ = 94.27 ± 5.20 fibers/1 mm^2^, AVG_Aged =_ 54.42 ± 5.51 fibers/1 mm^2,^ *p* = 0.049535; Central AN: AVG_Young_ = 91.64 ± 17.37 fibers/1 mm^2^, AVG_Aged =_ 69.43 ± 5.11 fibers/1 mm^2^, *p* = 0.12663) (**Figure 7L**; **Table 3**). The age-related decrease in AN fiber density is also apparent in longitudinal sections (**Supplementary Figure 5**).

**Table 3:** Average auditory nerve fiber density (# per 1 mm^2^), axon diameter (µm), myelinated fiber diameter (µm), g-ratio, and myelin sheath thickness (µm) in young and aged CBA/CaJ mice by peripheral and central auditory nerve region.

	Age	Whole AN	Peripheral AN	Central AN	Peripheral vs Central Mann-Whitney
		(range)	(range)	(range)	p-value
Fiber Density	Young	92.96 (72.97 - 100.79)	94.27 (88.07 - 100.79)	91.64 (72.97 - 114.80)	0.51
	Aged	61.93 (50.17 - 74.63)	54.42 (50.17 - 62.20)	69.43 (71.18 - 74.63)	4.95 x 10^-2^
	Young vs Aged Mann-Whitney				
	p-value	6.49 x 10^-3^	4.95 x 10^-2^	0.12663	
Axon Diameter	Young	55.67 (52.93 - 58.72)	56.50 (54.28 - 58.72)	54.84 (52.93 - 56.75)	0.43
	Aged	64.45 (62.19 - 67.28)	65.57 (63.86 - 67.28)	63.33 (62.19 - 64.48)	0.44
	Young vs Aged Mann-Whitney				
	p-value	2.09 x 10^-2^	0.12	0.12	
Myelinated Fiber Diameter (Axon + Myelin)	Young	102.41 (93.63 - 108.78)	105.41 (102.05 - 108.78)	99.40 (93.63 - 105.17)	0.44
	Aged	106.49 (101.98 - 111.33)	106.32 (106.17 - 106.46)	106.66 (101.98 - 111.33)	1.00
	Young vs Aged Mann-Whitney				
	p-value	0.39	1.00	0.44	
G-ratio	Young	0.54 (0.53 - 0.56)	0.53 (0.53 - 0.54)	0.55 (0.54 - 0.56)	0.25
	Aged	0.60 (0.57 - 0.63)	0.61 (0.59 - 0.63)	0.59 (0.57 - 0.60)	0.44
	Young vs Aged Mann-Whitney				
	p-value	2.09 x 10^-2^	0.12	0.12	
Myelin Sheath Thickness	Young	23.37 (20.35 - 25.03)	24.46 (23.89 - 25.03)	22.28 (20.35 - 24.21)	0.44
	Aged	21.02 (19.44 - 23.43)	20.37 (19.90 - 23.43)	21.66 (19.90 - 23.43)	0.44
	Young vs Aged Mann-Whitney				
	p-value	8.30 x 10^-2^	0.12	0.12	

### 3.4 AN fiber size distributions shift toward larger-diameter axons with thinner relative myelin

**Figure 8:**
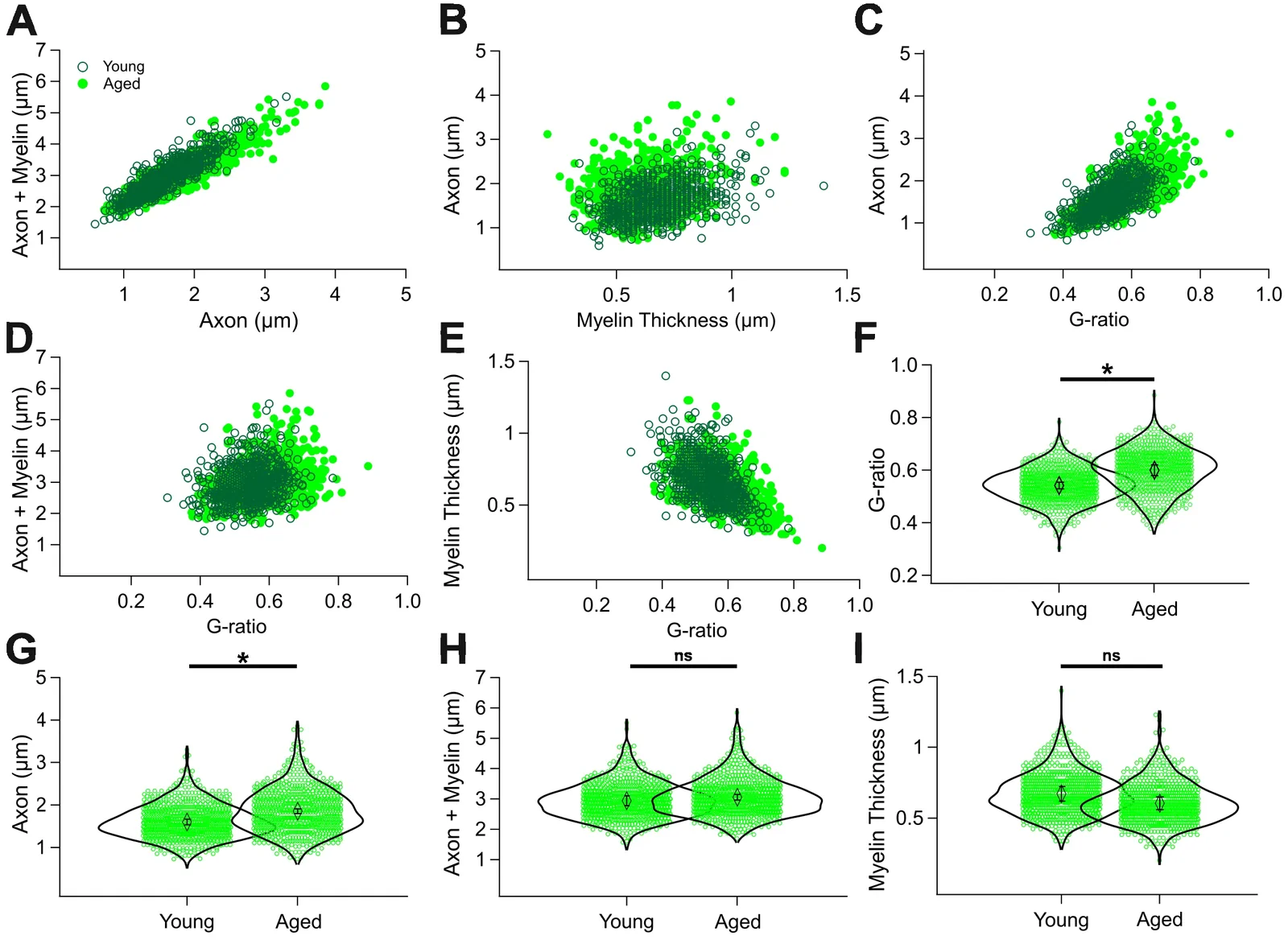
Aging is associated with increased AN g-ratio and larger mean axon diameter. **(A–E)** Distributions of axon diameter, axon + myelin diameter, myelin thickness, and g-ratio in young and aged mice. N = 4 mice per group with 8 ROIs per animal (4 peripheral and 4 central). Young = 813 Fibers; Aged = 800 Fibers. **(F–I)** Violin Plots of the g-ratio, axon diameter, axon + myelin diameter, and myelin thickness. Individual fibers are indicated by the open green dots. All distributions were evaluated for normality using a Kolmogorov-Smirnoff test. Significance was determined using a Mann-Whitney U-test, α = 0.05. Diamonds show the mean, and error bars represent the standard deviation. *p < 0.05.

Age-related changes in myelination of the AN were further quantified using Toluidine Blue stained sections. We manually measured axon diameter and myelinated fiber diameter (axon + myelin) of AN fibers in young and aged mice. We then calculated the g-ratios and thickness of the myelin sheaths in young and aged mice, including the peripheral and central AN. Analysis revealed age-related changes in the distribution of AN fiber size in CBA/CaJ mice (**Figures 8A–E**). G-ratios were significantly greater in aged mice compared to young (**Figure 8F**) (AVG_Young_ = 0.54 ± 0.01 µm, AVG_Aged_ = 0.60 ± 0.02 µm, *p* = 0.021). AN fibers in aged mice tended to have larger axon diameters compared to young (**Figure 8G**) (AVG_Young_ = 55.67 ± 2.23 µm, AVG_Aged_ = 64.45 ± 1.84 µm, *p* = 0.021). Measurements of myelinated fiber diameters showed no significant difference (**Figure 8H**) (AVG_Young_ = 102.41 ± 5.60 µm, AVG_Aged_ = 106.49 ± 3.31 µm, *p* = 0.39). Average thickness of the myelin sheath was not significantly different in aged mice compared to young (**Figure 8I**; **Table 3**) (AVG_Young_ = 23.37 ± 1.79 µm, AVG_Aged_ = 21.02 ± 1.55 µm, *p* = 0.083).

### 3.5 Aging alters AN gene expression of myelination and immune activation pathways

**Figure 9:**
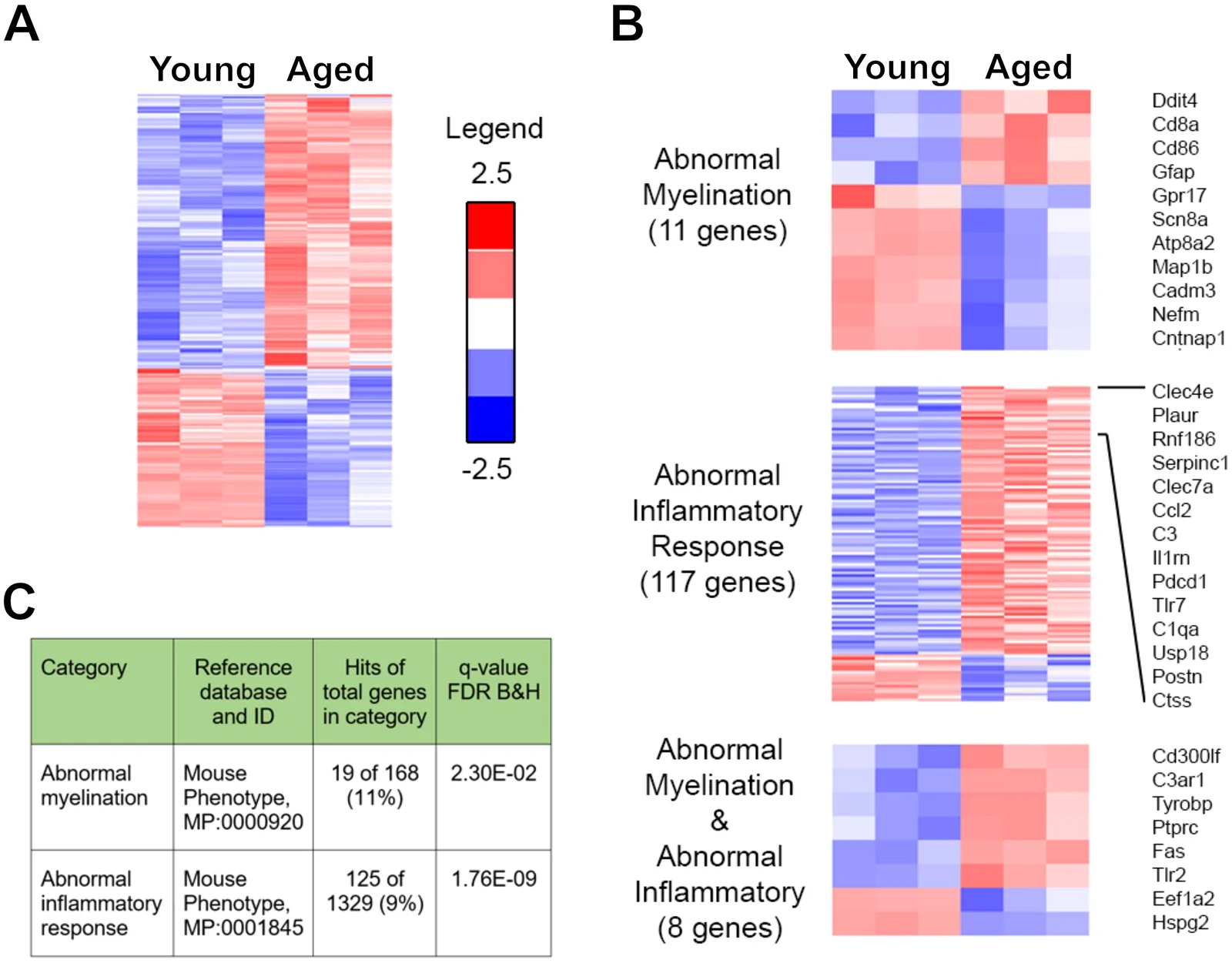
Gene expression profiles of the AN revealed age-dependent effects on inflammation and myelination. **(A)** Analysis of AN RNA-sequencing data from young (3 months) and aged (2.5 years) CBA/CaJ mice identified 1240 differentially expressed genes (**Supplementary Table 1**). **(B)** Expression patterns of differentially expressed genes linked to abnormal myelination, abnormal inflammatory response, or both categories. For abnormal inflammatory response, the 15 genes showing highest upregulation are listed on the right. **(C)** Enrichment analysis of differentially expressed genes identified significant age-related effects on abnormal myelination and abnormal inflammatory response.

To further validate our observations of age-related myelin degeneration and increased immune cell activity within the AN, bulk RNA-sequencing data obtained from a prior study of AN from young adult mice and aged mice was analyzed anew, using updated reference databases. Samples in the study contained central AN and peripheral AN within the cochlear modiolus and internal auditory meatus (IAM). Analysis of the RNA-sequencing data revealed significant age-dependent transcriptional changes in 1240 genes (FDR < 0.05; absolute fold change > 2) (**Figure 9A**). Functional enrichment analysis of these genes detected significant effects on pathways relating to myelination and immune activation, including abnormal myelination (q = 2.30 x 10^-2^) and abnormal inflammatory response (q = 1.76 x 10^-9^) (**Figures 9B, C**). Significantly upregulated genes mapping to these pathways included *Clec7a*, *Trem2*, and *Itgb2* (abnormal inflammatory response). A complete list of differentially expressed genes, and their association(s) with these pathways, is provided in **Supplementary Table 1**. Detection of an effect on abnormal myelination aligns with our observed immunohistochemistry results in the aged mouse AN. Together, these findings suggest that aging is associated with dysregulation of myelin-associated processes and inflammatory signaling.

### 3.6 Accumulation of myelin debris was found in macrophages/microglia populating the aged AN

**Figure 10:**
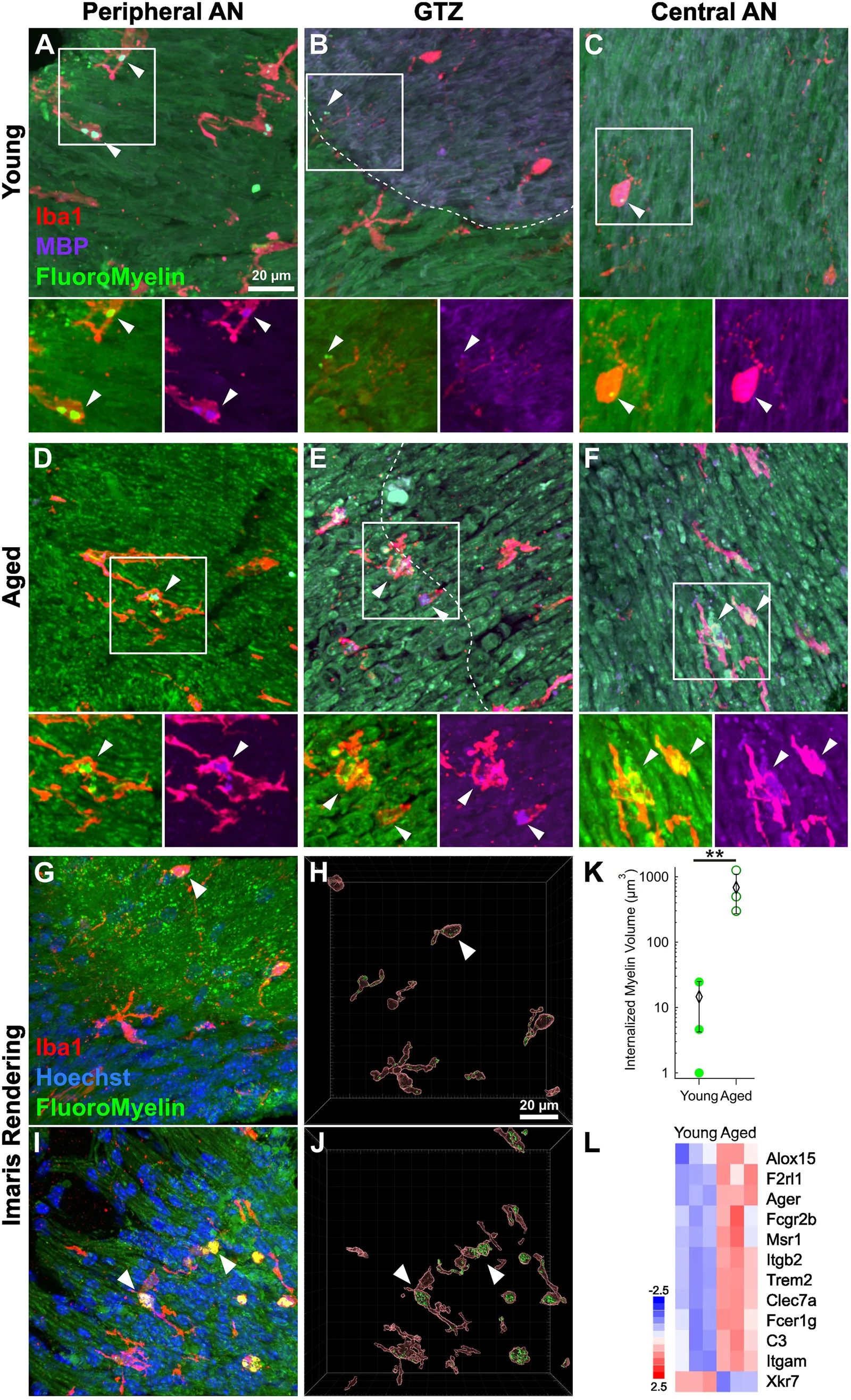
Increased internalized myelin debris and upregulated expression of phagocytosis-related genes appeared in the aged AN. **(A–F)** Sections of the peripheral AN **(A, D)**, AN GTZ **(B, E)**, and central AN **(C, F)** showing myelin debris, labeled by FluoroMyelin™ and MBP, within Iba1^+^ cells. **(G–J)** Representative images showing internalized myelin debris in young **(G)** and aged **(I)** mice, with corresponding Imaris three-dimensional reconstructions shown in **(H, J)**, respectively. **(K)** Quantification of internalized myelin volume in young and aged mice. N = 3 mice per age group, with 4 ROIs analyzed per mouse. **(L)** Differentially expressed genes linked to phagocytic engulfment are predominantly upregulated in aged (2.5 years) compared with young (3 months) mice. All distributions were assessed for normality using a Kolmogorov-Smirnoff test. Statistical significance between young and aged was determined using a Mann-Whitney U test, α = 0.05. **p < 0.01.

Given the proven function of macrophages/microglia in phagocytic processes associated with myelin maintenance, we evaluated this process in aging Iba1^+^ cells (16, 17, 22, 59, 71–75). Upon close examination of the Iba1^+^ cells within the AN, many contained internalized myelin debris, especially in the aged ANs (**Figure 10**). The increased presence of Iba1^+^ cells burdened with internalized myelin suggests possible immune dysfunction and dysregulation of the myelination process (59, 74, 76, 77). Young mice had very little internalized myelin debris within Iba1^+^ cells (**Figures 10A–C**), while aged mice had large amounts of internalized myelin debris within their cell bodies (**Figures 10D–F**). The presence of myelin withi Iba1^+^ cells in the AN is confirmed by its dual labeling with FluoroMyelin™ and MBP. This is evident in the immunofluorescence images, as well as in the Imaris reconstructions (**Figures 10G–J**). These age-related differences were consistent across the peripheral, central, and GTZ regions of the AN. Quantifying the volume of internalized myelin contained within Iba1^+^ cells, aged mice had significantly more internalized myelin compared to young mice (Internalized Myelin Volume: AVG_Young_ = 9.80 ± 10.69 µm^3^, AVG_Aged_ = 686.71 ± 414.09 µm^3^, *p* = 0.004) (**Figure 10K**). To further explore the effects of aging on phagocytic functions, our bulk RNA-sequencing results were re-examined with the finding that phagocytic categories were significantly enriched in the differentially expressed gene set, including “phagocytosis, engulfment” (GO:0006911; q = 3.67 x 10^-4^). Review of expression patterns for genes related to phagocytic engulfment showed that they were predominantly upregulated in aged AN (**Figure 10L**).

### 3.7 Myelin phagocytosis by Iba1^+^ cells is increased at the AN GTZ with aging

**Figure 11:**
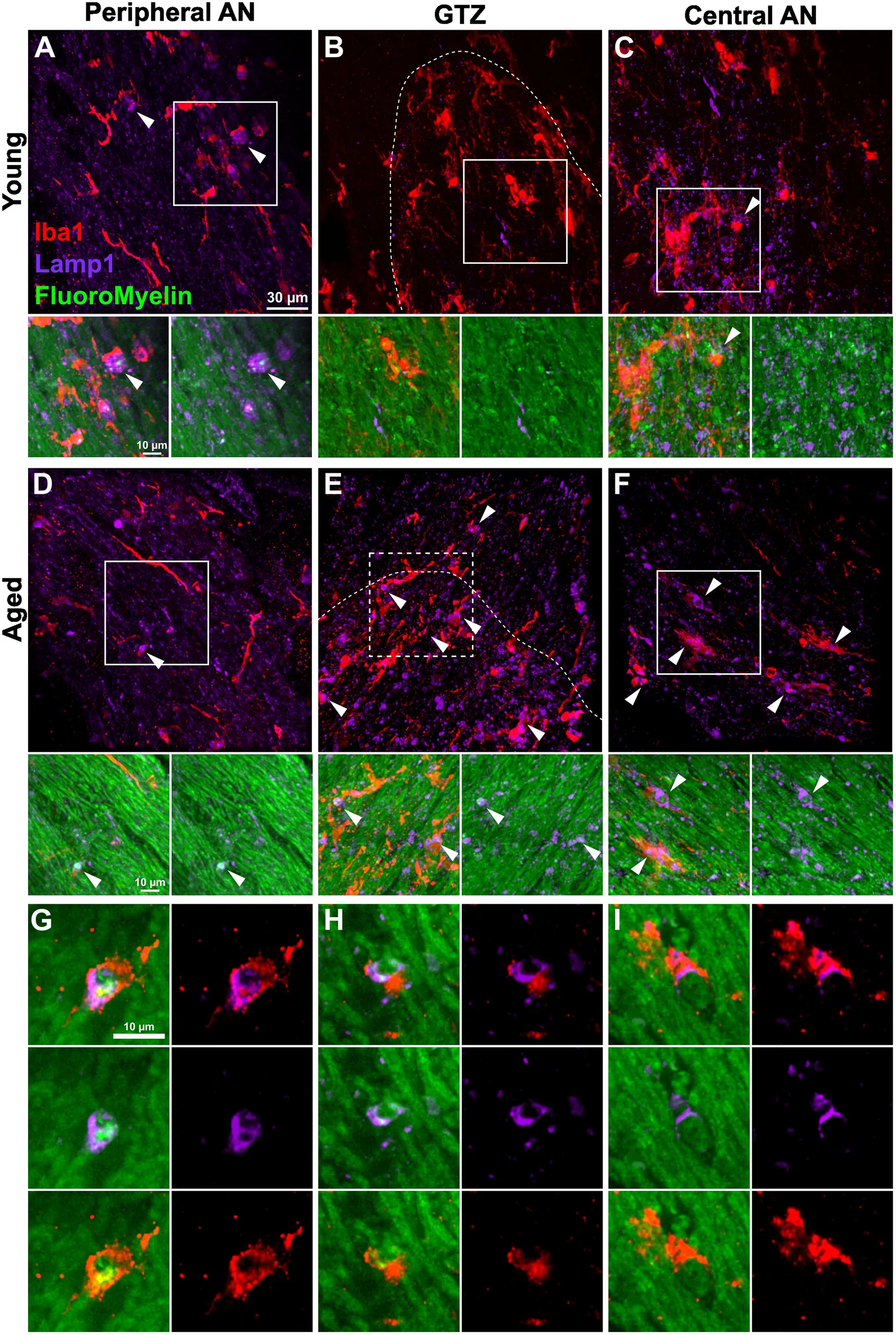
Lamp1^+^ Iba1^+^ cells at the aged AN GTZ indicate increased phagocytic activity. **(A–F)** Sections of the peripheral AN **(A, D)** AN GTZ **(B, E)**, and central AN **(C, F)** showing Lamp1^+^ and Iba1^+^ cells in the AN. White boxes indicate regions shown at higher magnification in the images below. White arrows indicate dual-labeled Iba1^+^ Lamp1^+^ cells. White dashed lines indicate the location of the AN GTZ with the peripheral AN at the top of each image. **(G–I)** Representative z-slices showing Lamp1^+^ Iba1^+^ cells with myelin inside Lamp1^+^ lysosomes in the aged AN GTZ. Sections were stained with FluoroMyelin™ (green), Iba1 (red), and Lamp1 (violet).

To assess if Iba1^+^ cells were engaged in active or excessive phagocytic processing of myelin debris, we stained for Lamp1, an established lysosomal marker of phagosomal processing (78–80). Young mice showed some Lamp1^+^ Iba1^+^ cells in the peripheral AN and central AN, with diffuse labeling of Lamp1 by other cell types in the AN, while the AN GTZ had little to no Lamp1^+^ Iba1^+^ cells (**Figures 11A–C**). In aged mice, Lamp1^+^ Iba1^+^ cells are also found throughout the central and peripheral AN, but we observed robust Lamp1 staining at the AN GTZ with more Lamp1^+^ Iba1^+^ cells at the AN GTZ with age (**Figures 11D–F**). Lamp1^+^ lysosomes within Iba1^+^ cells are often identified containing and engulfing myelin near the AN GTZ (**Figures 11G–I**).

### 3.8 Iba1^+^ cells at the AN GTZ contain normal-appearing myelin

**Figure 12:**
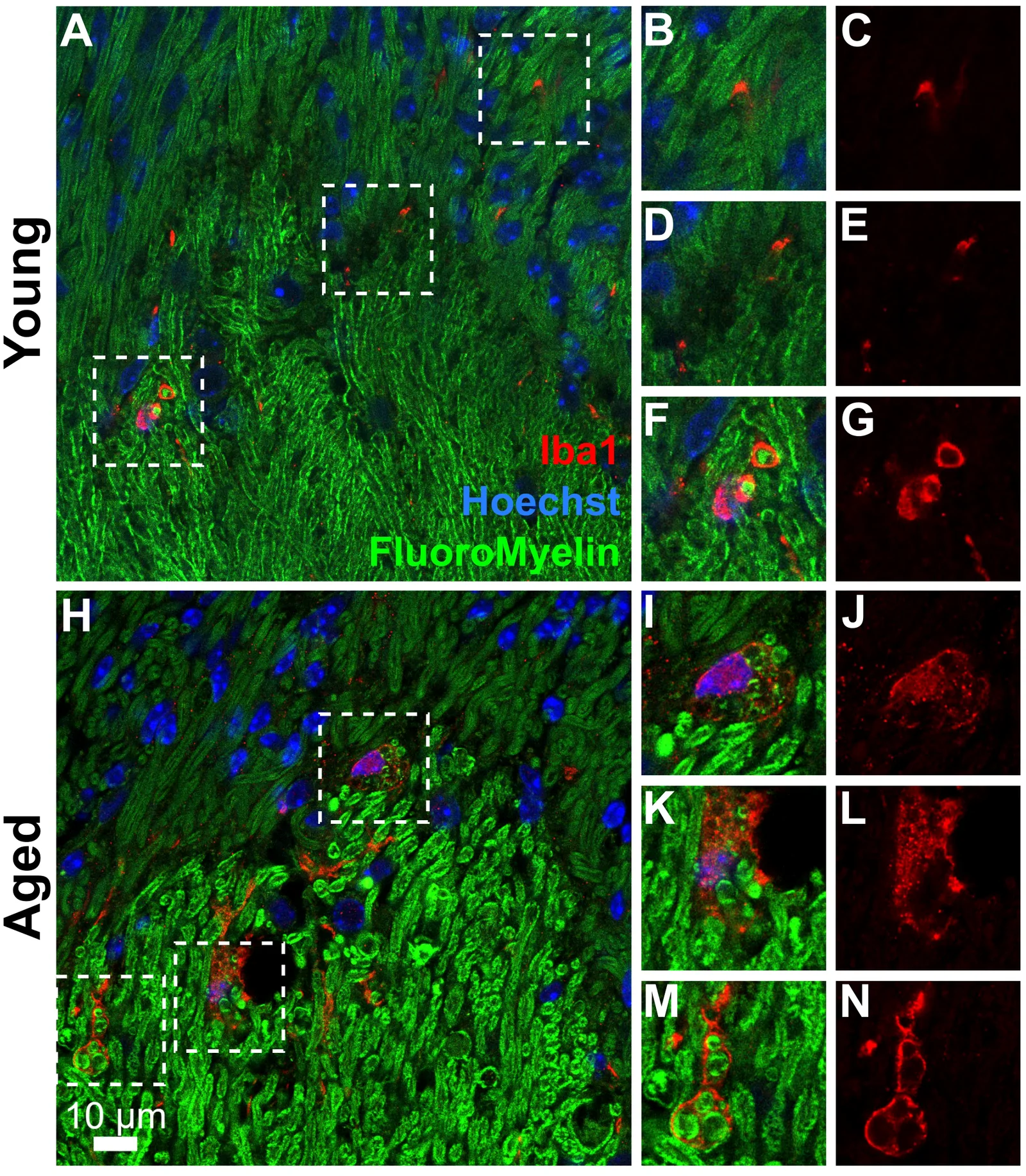
Iba1^+^ cells at the aging AN GTZ contain structurally intact myelin sheaths. **(A–N)** Sections of the AN GTZ from young **(A–G)** and aged **(H–N)** mice showing Iba1^+^ cells with internalized myelin. Dotted boxes in A and H indicate regions enlarged in **(B–G, I–N)**, respectively, to show structurally intact myelin within Iba1^+^ macrophages/microglia at the AN GTZ. Images are individual slices from the z-stack to show the structurally intact myelin within Iba1^+^ cells.

Examining morphological differences in the internalized myelin within Iba1^+^ macrophages/microglia in the peripheral AN, central AN, and the AN GTZ reveals a unique morphology of internalized myelin found only in the aging AN GTZ. Young mice had very few Iba1^+^ cells with any internalized myelin across regions of the AN, including the AN GTZ (**Figures 12A–G**). By contrast, aged mice had numerous Iba1^+^ cells with internalized myelin debris throughout the AN, but the AN GTZ macrophages/microglia contained myelin that appeared structurally intact (**Figures 12H–N**). Upon close inspection of the higher magnification images, there are Iba1^+^ cell bodies containing normal-appearing myelin (**Figure 12**). Additionally, the morphology of these internalized FluoroMyelin™-stained structures have the appearance of an open cylinder, which is different from the disorganized appearance of myelin debris shown in **Figures 10**, **11**. This specific feature suggests that the phagocytic processes performed by immune cells at the GTZ involve both myelin debris and normal-appearing myelin of aged AN fibers.

### 3.9 Human temporal bones have Iba1^+^ cells with internalized myelin throughout the AN

**Figure 13:**
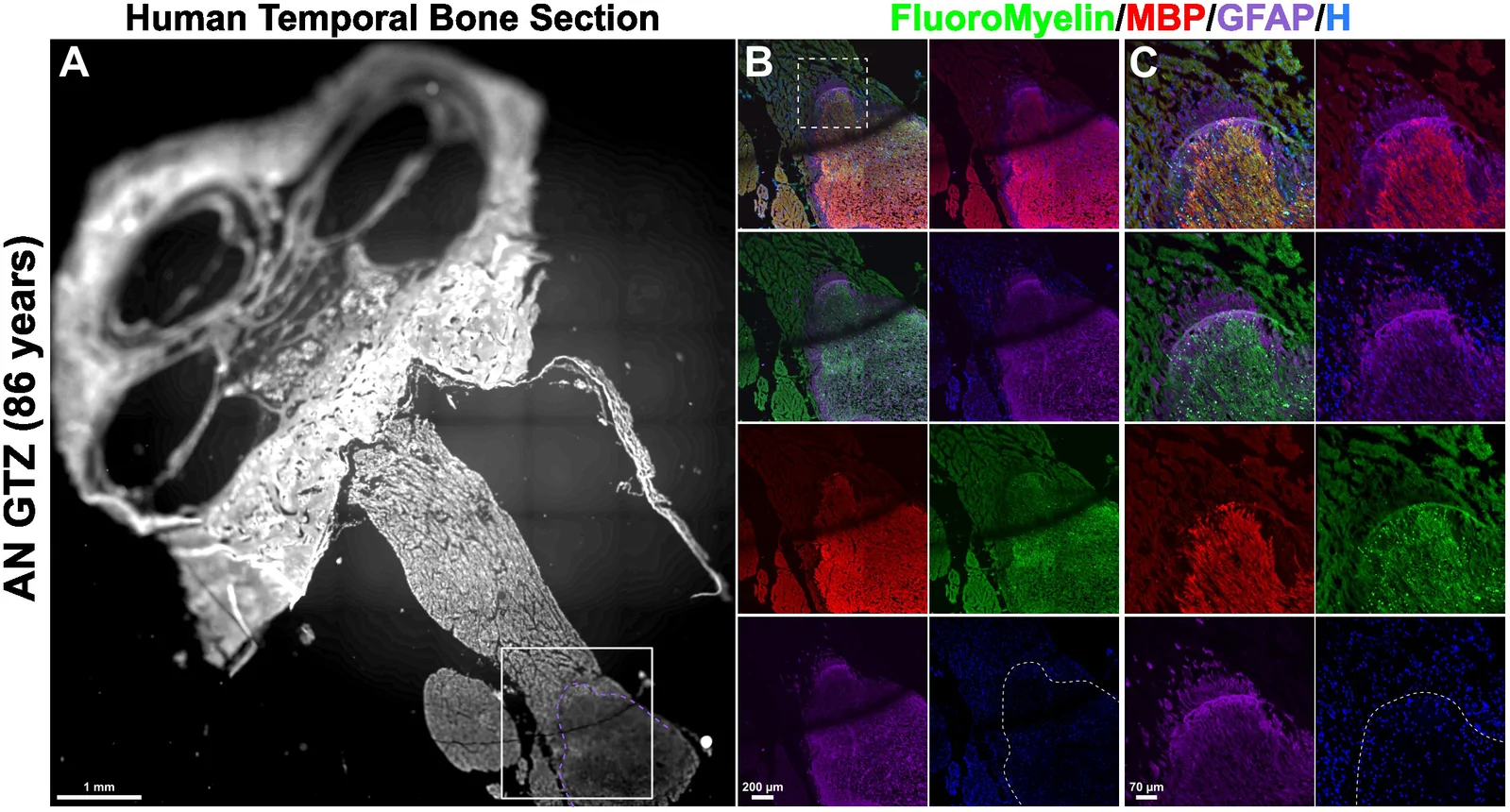
Human AN shows central-peripheral differences in myelin composition across the GTZ. **(A)** Low magnification image of a whole temporal bone section from an 86-year-old donor. Violet dashed line indicates the location of the AN GTZ housed in the internal auditory meatus (IAM). White box indicates the location of the magnified region **(B)**. Immunostained sections of the AN GTZ with the peripheral AN oriented at the top of each image. Dashed box indicates the location of the magnified region in **(C)**. Magnified views of the AN GTZ from the same donor showing FluoroMyelin™ and MBP labeled myelin sheaths in both the peripheral and central AN, with differing staining intensities consistent with regional differences in myelin composition. GFAP labeling also shows the boundary between central and peripheral. White dashed lines in B and C indicate the location of the AN GTZ. Sections are stained with FluoroMyelin™ (green), MBP (red), GFAP (violet), and Hoechst (blue).

**Figure 14:**
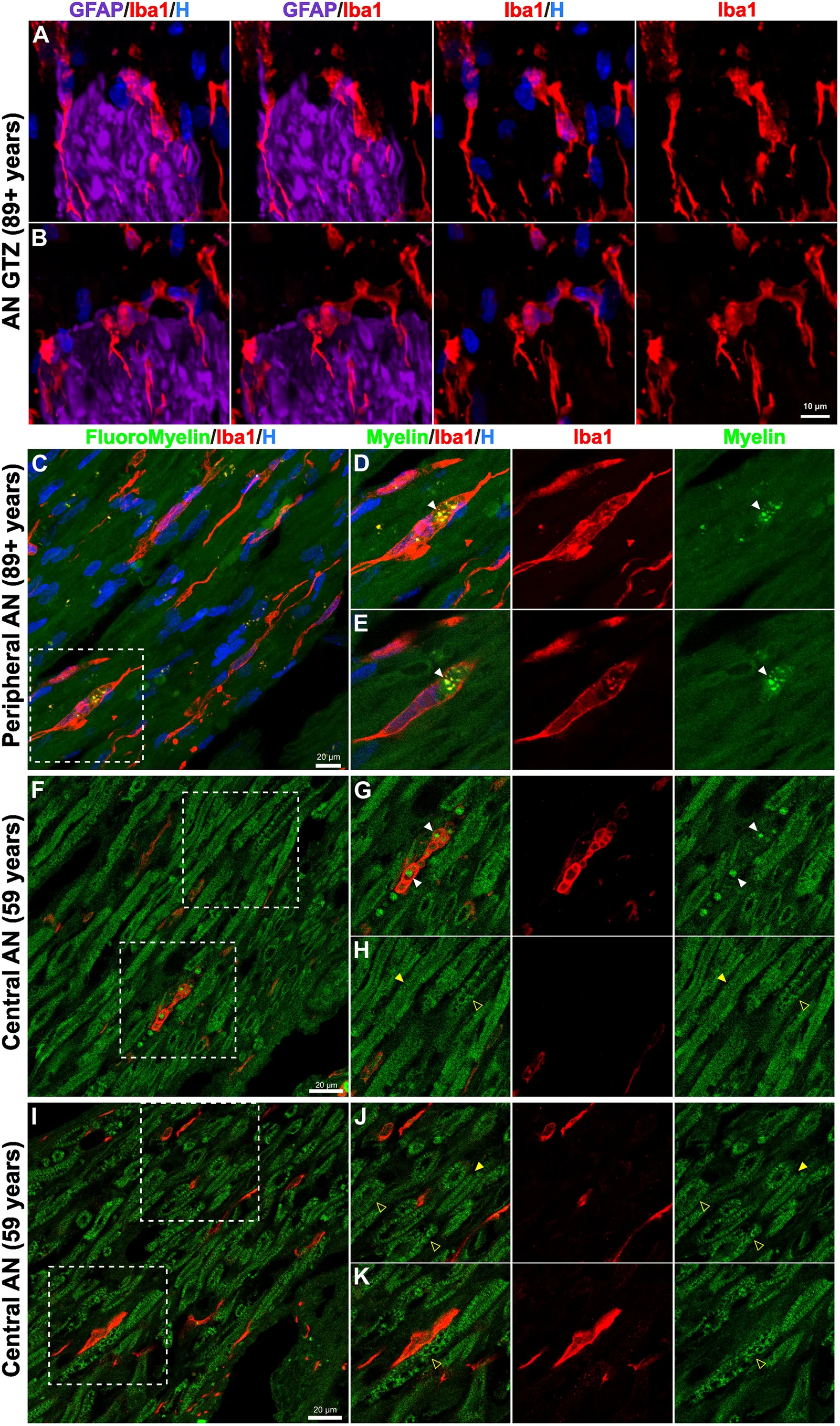
Internalized myelin found in human AN Iba1^+^ cells. **(A, B)** Sections of the AN GTZ, indicated by the GFAP staining (violet), in a temporal bone from an 89+ year old donor showing Iba1^+^ cells in the region. **(C)** Immunostained section of the peripheral AN in an 89+ year old donor demonstrating internalized myelin within an Iba1^+^ cell. **(D)** Enlarged view of the dashed white box in **(C)**. White arrows indicate internalized myelin debris. **(E)** Mid-stack Z-slice from the image in **D** demonstrating myelin within an Iba1^+^ cell. **(F–K)** Immunostained sections of the central AN in a donor (59 years old) showing myelin within an Iba1^+^ cell **(G)**, variability in myelin architecture consistent with age-related pathology **(H–J)**, and close association of Iba1^+^ cells with regions of myelin disruption **(K)**. Solid yellow arrows indicate normal myelin and open yellow arrows indicate disrupted myelin. Sections were stained with FluoroMyelin™ (green), Iba1 (red), GFAP (violet), and Hoechst (blue).

To determine if similar age-related changes in immune-myelin interactions in mice were also present in the human AN, we analyzed human temporal bone sections at the AN GTZ. We used sections of the temporal bones from aged donors (59, 86, and 89+ years) to look at aging effects on the AN. Locating the appropriate sections from human temporal bones is challenging, and identifying the specific sections that contain the GTZ is difficult due to the large number of sections (>200 per specimen), as well as the exact angle required to capture the intact AN GTZ housed within the IAM. Once we located the appropriate human temporal bone sections, sections were immunostained and then imaged to evaluate the AN GTZ (**Figure 13**) and Iba1^+^ macrophages/microglia present at the peripheral AN, central AN, and AN GTZ (**Figure 14**). A low magnification image of the human temporal bone section shows the location of the AN GTZ within the IAM and greater than 5mm from the basal turn of the cochlea (**Figure 13A**). The boundary between the central and peripheral AN was identified by glial fibrillary acidic protein (GFAP) immunostaining, which labeled astrocytes at the boundary and within the central AN (**Figures 13B, C**). The difference in staining intensity of MBP between the central AN and peripheral AN (**Figures 13B, C**) is also apparent in human temporal bones. FluoroMyelin™ and MBP both stain the myelinated fibers of the AN (**Figure 13B**). Examining the AN GTZ, we can see the distinct boundary between the peripheral and central myelinated regions of the AN (**Figure 13C**).

We identified Iba1^+^ cells throughout the human AN from three donors (**Figure 14**). The GTZ was also identifiable in human sections based on the difference in density of nuclear staining (more nuclei peripherally due to myelination by Schwann cells and fewer nuclei centrally due to myelination by oligodendrocytes) (**Figures 13B, C**). Consistent with our findings in the mouse AN GTZ, we observed Iba1^+^ cells distributed along the human AN GTZ (**Figures 14A, B**). High-resolution imaging revealed Iba1^+^ cells in direct contact with myelin sheaths of the AN both centrally and peripherally (**Figures 14C–H**). Iba1^+^ cells containing internalized myelin debris were also observed within the peripheral AN (**Figures 14C–E**) and central AN (**Figures 14F–K**), suggesting phagocytic processes occur in the aging human AN. We also observed variability in myelin integrity in the AN, with some fibers tightly sheathed and others showing evidence of myelin disruption (**Figures 14H–K**). Additionally, some Iba1^+^ cells were observed in close association with disrupted myelin sheaths (**Figures 14I–K**). These findings suggest that interactions between Iba1^+^ macrophages/microglia and myelinating glia may be relevant to age-related changes in the human AN.

## 4 Discussion

The present study identifies the AN GTZ as a central-peripheral neuroimmune interface with age-related alterations in AN myelination and immune cell activity, both of which may contribute to AN degeneration and functional declines associated with ARHL. By integrating large-field light sheet microscopy of tissue cleared mouse temporal bones, three-dimensional high-resolution imaging, quantitative immunohistochemistry, RNA-sequencing, and human temporal bone studies, we demonstrate that this central-peripheral interface is not just a boundary where myelination shifts, but a dynamic region characterized by age-related immune activation and increased phagocytic activity within the AN. Consistent with previous studies of age-related AN degeneration (26, 40, 50, 81), our findings also suggest that aging is associated with myelin degeneration and relative myelin thinning among the surviving large-diameter axons.

**Figure 15:**
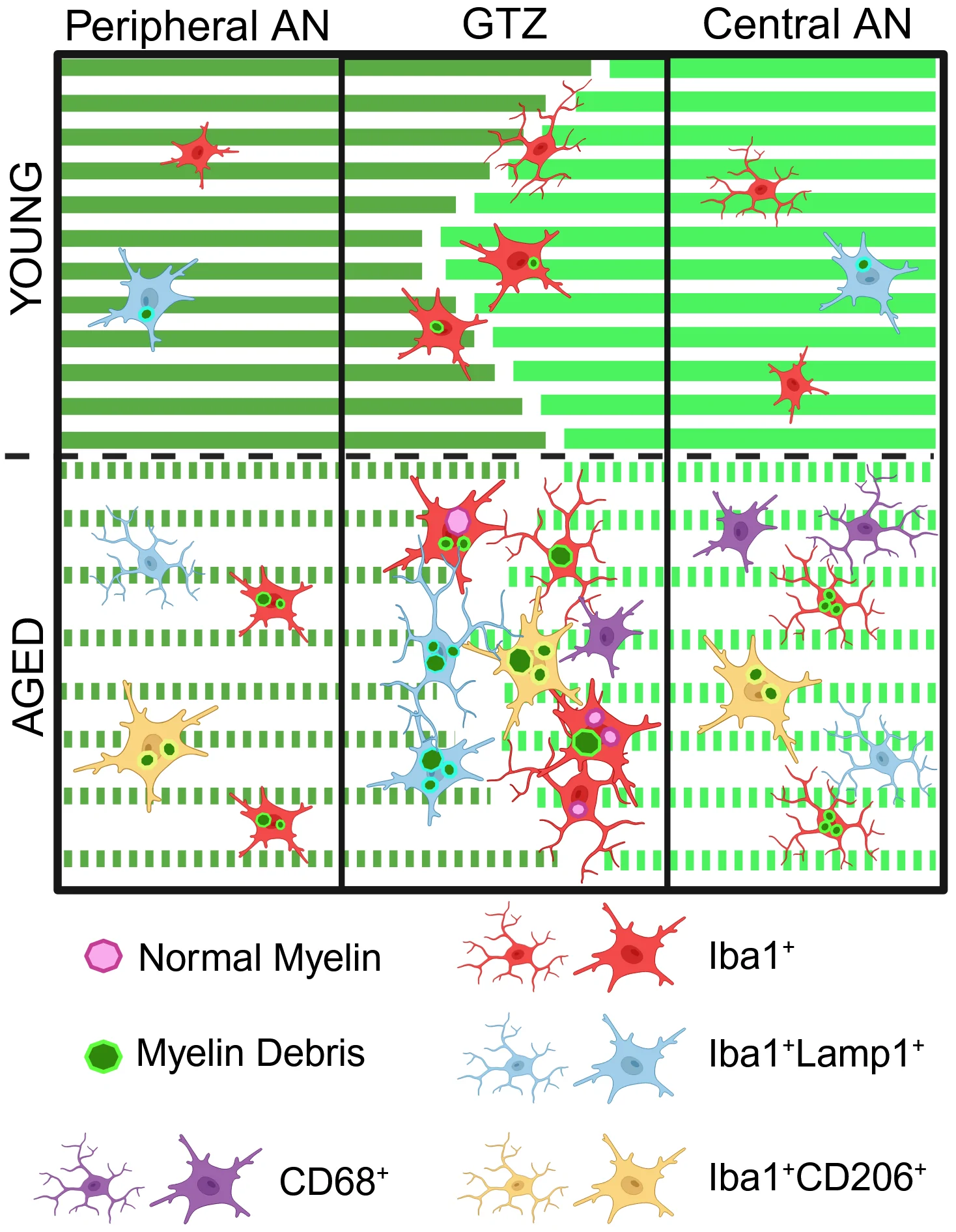
Summary of Aging Effects on the AN and AN GTZ. Schematic summarizing age-related changes in the AN and AN GTZ. Aging was associated with decreased AN fiber density and reduced relative myelination, together with an increased number of Iba1^+^ macrophages/microglia throughout the AN, with accumulation at the AN GTZ. CD68^+^ cells were increased in the aged central AN and AN GTZ. Iba1^+^CD206^+^ were increased in aging and contained larger intracellular CD206 inclusions. Iba1^+^Lamp1^+^ cells were present at the AN GTZ in aging, but absent in young controls, suggesting enhanced phagocytic activity. While Iba1^+^ cells containing myelin debris were found throughout the aged AN, the GTZ exhibited a marked increase in Iba1^+^ cells containing normal myelin, consistent with GTZ-specific age-related immune activation and myelin phagocytosis.

Aged ANs showed a substantial increase in Iba1^+^ cells and CD68^+^ cells, with accumulation at the AN GTZ. In the aged AN, subsets of Iba1^+^ cells also co-labeled with CD206, Gal3, CD163, or Lamp1, with CD206^+^ Lamp1^+^ and CD163^+^ Iba1^+^ cells detected at the AN GTZ. These findings highlight the heterogeneity of immune cell states present in the aging AN (**Figure 15**). Additionally, Iba1^+^ cells contained increased internalized myelin debris throughout the aged AN and normal-appearing myelin was frequently observed within Iba1^+^ cells at the AN GTZ. Consistent with our findings in mice, human temporal bone analyses indicate central-peripheral differences in myelin composition and demonstrated internalized myelin within Iba1^+^ cells throughout the human AN. Together, these findings position the AN GTZ as a unique neuroimmune microenvironment with age-related alterations in phagocytic activity and immune activation. Defining GTZ-associated immune cell subpopulations and their altered immune states may provide insight into mechanisms contributing to age-related changes in the AN and help identify pathways relevant to ARHL. Additionally, these findings within the AN may also offer insight into the biology of GTZs throughout the nervous system.

### 4.1 The AN GTZ is a unique biological niche with age-related alterations in immune activity

Previous studies note the existence of GTZ regions and their development across spinal and cranial nerves (7, 82–85). In the auditory system, coordinated migration of oligodendrocytes at the AN GTZ during development creates a border between peripheral and central in cat, rat, and mouse models (8–10). Our findings extend these observations by better defining the adult AN GTZ as an immune-active central-peripheral interface located within the cochlear modiolus in mice and within the IAM in humans.

Our study offers a new approach for studying the cochlear macrophage/microglia subpopulations and their altered immune states within the AN across both the central and peripheral nervous systems. The AN GTZ contains both the central and peripheral glial microenvironments housed within the cochlea. Our data revealed that the AN GTZ contains a sharply demarcated shift in myelination between the peripheral and central AN regions, with FluoroMyelin™ and MBP staining supporting regional differences between central and peripheral myelin composition (**Figures 1**–**3**). NG2 staining further supported the distinction between central and peripheral glial environments, with NG2^+^ somas enriched centrally and more diffuse NG2 labeling observed peripherally. In contrast to the clear myelin boundary, the myeloid compartment is far less segregated in the GTZ (**Figures 4**–**6**). At the AN GTZ, the processes of some Iba1^+^ cells extend to both the peripheral and central AN (**Figures 4**–**6**), suggesting that macrophages/microglia may interact and move freely across this boundary and that the AN GTZ may support communication between central and peripheral.

With aging, the AN GTZ showed prominent immune cell accumulation. Whole mouse temporal bone light sheet imaging revealed more CD68^+^ cells in the aged ANs and accumulation at the AN GTZ (**Figures 1**, **2**). Iba1^+^ cells were also increased throughout the aged AN, with the GTZ showing one of the most prominent regional increases (**Figure 5**). In addition, CD206^+^, Gal3^+^, CD163^+^, and Lamp1^+^ immune cell populations were identified in the aged AN, with enrichment of Lamp1^+^ Iba1^+^ cells and CD206^+^ Iba1^+^ cells at the AN GTZ (**Figures 6**, **11**; **Supplementary Figures 3**, **4**). These findings suggest that aging is associated with increased immune cell heterogeneity within the AN and that the AN GTZ may serve as a focal site for age-related immune-myelin interactions at the interface between the central and peripheral AN.

This central-peripheral interface located within the cochlear modiolus may represent a potential immunological niche where macrophages and microglia coexist or converge to coordinate their responses to age-related pathology. Notably, there is no physical barrier separating the peripheral and central immune microenvironments; rather, the GTZ is defined by a discrete gap in myelination where the shift between Schwann cells and oligodendrocytes occurs (7, 10) (**Figures 3**, **5**, **7**, **8**, **12**, **13**). One study reported that glia can adapt and change their phenotype in response to dysfunctional neighboring glia (86). It has also been reported that microglia mount a specialized response at root entry zones in both the peripheral and central portions of the sciatic nerve after injury (87, 88). Additionally, studies have reported that the various glial cells, including oligodendrocytes, astrocytes, Schwann cells, and macrophages/microglia, have plasticity in phenotype in response to injury (89, 90). The age-related increase in Iba1^+^ cells and their increased co-labeling with CD206, Gal3, CD163, and Lamp1 suggest increased immune cell heterogeneity within the AN and may reflect altered immune cell states with aging.

### 4.2 Aging alters myelination of surviving large diameter AN fibers

A defining feature of the aging AN in our study was the coordinated emergence of structural changes in both AN fibers and associated myelin. Aging is associated with demyelination and altered myelin maintenance in the brain (91–93), but the effects of aging on AN myelin across the peripheral and central regions of the AN remain poorly defined. Our data showed that aging is associated with disrupted myelin sheath morphology in both the peripheral and central AN, reduced AN fiber density, and increased g-ratio, together supporting myelin degeneration. We also found an increase in mean axon diameter in the aged AN, suggesting a preferential loss of AN fibers with smaller diameter, which are likely low-spontaneous rate fibers based on studies in cat and mouse (94–96). Selective loss or reduced activity of low spontaneous rate fibers has been well documented in the aging AN (81, 97). These findings suggest that aging alters the organization and composition of the AN, with relative preservation of larger diameter AN fibers with associated myelin degeneration. However, future studies should perform morphometric analysis with a focus on variations by frequency location and AN fiber subtypes.

The age-related myelin phenotype was not simply explained by a uniform loss of myelin (**Figures 1**, **2**). Although aged ANs showed disrupted myelin sheaths and reduced fiber density, myelinated fiber surface area and volume were not significantly altered (**Figure 7**). The reduction in fiber density, particularly in the peripheral AN, further supports age-related structural degeneration of AN fibers, while the presence of myelin disruption in both peripheral and central regions indicates that age-related myelin pathology is not restricted to either compartment. These structural changes may create a permissive environment (23, 98) for altered immune activity and myelin processing by macrophages/microglia in the aging AN.

### 4.3 The aging AN GTZ exhibits altered phagocytic activity and engulfment of normal-appearing myelin

The aging AN showed increased immune activity and evidence of altered phagocytic engagement by Iba1^+^ cells at the AN GTZ. Aging has been linked with phagocytic dysfunction in the brain, especially in the context of neurodegenerative disease, some of which can cause hearing loss (20, 23, 25, 99–102). Although the presence of Iba1^+^ cells in the cochlea and AN has been previously described, their functions in age-related myelin pathology remain poorly understood (33, 103–106).

The aged AN GTZ showed increased Iba1^+^ cells and CD68^+^ cells with some expressing CD206, Gal3, CD163, and Lamp1, markers associated with activation, phagocytosis, and lysosomal processing. Some of these markers have also been identified in human otopathology studies (70). We found Iba1^+^ Lamp1^+^ cells at the AN GTZ with aging, but not at the AN GTZ in young mice (**Figure 11**). Additionally, the presence of myelin within Lamp1^+^ lysosomes supports increased phagocytic activity in the aged AN and heightened activity at the AN GTZ (**Figures 11**, **12**, **14**). High-resolution imaging revealed Iba1^+^ macrophages/microglia in aged mice contained myelin debris labeled by FluoroMyelin™ and MBP, suggesting active phagocytic engagement in the aged AN (**Figures 10**, **11**).

One of the most notable findings was frequent observation of normal-appearing myelin within Iba1^+^ cells at the aged AN GTZ (**Figure 12**). This was not observed in either the peripheral or central AN regions and suggests that the GTZ may have a distinct phagocytic microenvironment. Since the GTZ brings together the peripheral and central glial environments, macrophages and microglia in this region may receive signals from Schwann cells and oligodendrocytes that alter their phagocytic activity and inflammatory state, which may contribute to the focal increase in immune activity at the aging AN GTZ. Future studies should focus on identifying specific macrophage and microglia populations and characterizing their phagocytic activity using advanced omics and direct functional assays of phagocytic activity in the AN.

The significance of internalized myelin within Iba1^+^ cells remains unclear. Previous studies in the brain have reported that myelin-loaded microglia is evidence of phagocytic dysfunction, characteristic of a proinflammatory phenotype, and can exacerbate disease progression (18, 107–109). In the aging brain, deficits in phagocytic activity associated with myelin turnover and maintenance are often found in neurodegenerative and demyelinating diseases such as Alzheimer’s disease, multiple sclerosis, and amyotrophic lateral sclerosis (110–112). However, because internalized myelin may reflect either adaptive debris clearance or maladaptive phagocytic activity, future studies are needed to determine if these Iba1^+^ populations are protective, pathogenic, or functionally heterogenous in the aging AN.

Transcriptomic analysis of aged ANs supports alterations in phagocytosis by Iba1^+^ cells of the aged AN, which may suggest stalled or dysfunctional clearance rather than efficient debris removal. This dysfunctional clearance may be linked to the upregulation of *Clec7a*, *Trem2*, and *Itgb2* (**Figure 9**). These genes have been linked to dysregulation of phagocytic processes and are expressed by disease-associated microglia in the brain (101, 113–117). However, a limitation of our study is that bulk RNA sequencing of whole ANs did not allow regional resolution between the central AN, peripheral AN, and GTZ. Further studies focusing on heterogeneity of the Iba1^+^ cell populations in the different regions of the AN and cochlea should be performed. Advanced omics approaches, including single-cell RNA sequencing and spatial omics, will be important for defining the spatial organization of age-related changes in AN immune cells. Defining the functions and molecular signatures of these immune cell populations will be essential for understanding how different immune cell states contribute to AN degeneration, demyelination, and chronic inflammation associated with ARHL.

### 4.4 Dynamic immune-myelin interactions are conserved in the human AN

To evaluate whether our findings in the mouse AN were also present in the human AN, we examined temporal bone sections from three donors. Human AN sections demonstrated central-peripheral differences in FluoroMyelin™ and MBP staining across the GTZ, consistent with regional differences in myelin composition like those observed in the mouse AN (**Figure 13**). The human AN also contained Iba1^+^ cells closely associated with myelin sheaths and containing internalized myelin (**Figure 14**). These findings support macrophage/microglia engagement with myelin in the human AN and suggest that phagocytic processing by Iba1^+^ cells also may occur in the aging human AN.

The AN GTZ is located within the cochlear modiolus in mice, whereas in humans the AN GTZ is found more centrally within the IAM (118). Despite these anatomical differences in AN GTZ location (**Figure 13**), our findings suggest that a dynamic immune-myelin interactions may contribute to age-related changes in the human AN as well. Although we observed evidence of phagocytic activity within the AN of both mice and humans, important species-specific difference in AN myelination should be considered. For example, spiral ganglion neurons of the AN are extensively myelinated in mice, but fewer than 5% of spiral ganglion neurons are myelinated in humans (119–121). Lack of myelin around the majority of spiral ganglion neurons may indicate distinct immune cell-myelin interaction within the human AN. These species-specific differences in AN myelin organization may influence age-related adaptions and functions of the immune cell subpopulations that surveil and maintain AN myelin.

Microglia turnover in the human brain is a slow continuous process, making this slowly renewing population particularly vulnerable to pathologic disturbances like aging and demyelination (91). These age-related changes in immune cell states within the AN and AN GTZ raise the possibility that similar changes occur at other GTZs throughout the nervous system. Whether such age-related alterations in immune microenvironments influence susceptibly to other GTZ-associated pathologies, including increased rates of schwannoma in cranial nerve VIII compared to other cranial nerves, remains unknown and warrants further investigation (55, 122–124). Our findings support previous studies highlighting the robust and diverse immune populations present in the human cochlea (125–128). However, because human temporal bone analyses were observational and without young controls for comparison, future studies across a broader range of ages will be needed to determine how these interactions between Iba1^+^ cells and myelin change with age in humans and how they relate to hearing function.

Our findings raise the possibility that identifying and defining the roles of specific immune cell subpopulations within the aging AN may improve our understanding of age-related demyelination and AN degeneration. Preclinical models have shown that modulating glial subtype can have a protective role, particularly in neurodegenerative disease (98, 101, 116, 117, 129). Rather than broadly suppressing immune activity, future studies will need to define specific macrophage/microglia subpopulations within the AN and determine how altering their activity affects AN function and aging. These data raise the possibility that altering macrophage/microglia activation states could preserve AN myelination, slow AN degeneration, and potentially mitigate pathophysiological changes associated with ARHL.

## Data Availability

The datasets presented in this study can be found in online repositories. The names of the repository/repositories and accession number(s) can be found below: GSE141865 (GEO).
