# Supplementary material for: The auditory nerve glial transition zone is a focal site of age-related immune-myelin interactions
Source: Front Immunol. 2026 Aug 7;17:1757234. doi: 10.3389/fimmu.2026.1757234 (PMC13493311; doi:10.3389/fimmu.2026.1757234)
Supplement: Supplementary file 1 [file DataSheet1.docx]

*Supplementary Material*

**Supplementary Figure 1. Auditory brainstem response (ABR) thresholds of CBA/CaJ mice used in this study.** ABR wave I thresholds at 11.3 kHz for young (3­–4 months) and aged (<2.5 years) mice of both sexes used in this study. Red markers indicate the young mice (filled circles; n=7) and aged mice (open circles; n=7). Means are indicated by black diamonds. Error bars indicate the standard deviation for each group.

**Supplementary Figure 2. Stacked serial sections of the mouse AN GTZ identifying more CD68^+^ cells in aged mice. (A–X)** Serial sections 25 μm apart from tissue-cleared mouse temporal bones, imaged via large-field light sheet microscopy, showing the entire AN within the cochlea and the AN GTZ in young (3 months) (**A–L**) and aged (2.5 years) (**M–X**) CBA/CaJ mice. **(D’, E’, I’)** Enlarged images of the dashed white regions of the AN GTZ in a young mouse are shown. **(P’, T’, V’)** Enlarged images of the white outlined regions of the AN GTZ in an aged mouse are shown with a white arrow indicating CD68^+^ cells within the AN, while few CD68^+^ cells were seen in the AN of the young mouse (**D’, E’, I’**). Dashed white lines in **D’, E’, I’**, and **P’** show the cone-shaped morphology of the AN GTZ. Dashed white lines in **T’** and **V’** show the GTZ in the basal turn/hook of the cochlea. Each mouse temporal bone was stained with MBP (green) and CD68 (red). Scale bars = 200 µm for all images.

**Supplementary Figure 3. CD163 is present at the aged AN GTZ. (A–H)** Twelve-micron sections of the AN in a young and old CBA/CaJ mouse. Sections were stained with FluoroMyelin^TM^ (green), Hoechst (H; cyan), Iba1 (red), and pro-inflammatory macrophage/microglia cell marker CD163 (violet). The white dashed line indicates the location of the AN GTZ (**A, E, I, M**). Scale bars = 20 µm for all images.

**Supplementary Figure 4. More Gal3^+^ observed within the aged AN. (A–F)** Immunostained sections of the AN in a young and aged CBA/CaJ mice. Sections were stained with Iba1 (red) and pro-inflammatory macrophage/microglia cell marker Gal3 (green). White dashed boxes indicate regions of magnification. White arrows indicate cells that are Gal3^+^ in the AN, with many found throughout the old mouse. Scale bars = 20 µm.

**Supplementary Figure 5. AN GTZ in longitudinal section shows gap between peripheral and central with aging. (A–B)** Epoxy resin embedded sections stained with Toluidine Blue demonstrating the age-related changes in the AN GTZ in young (**A**) and aged (**B**) mice. Red dashed lines indicate the GTZ with the peripheral AN at the top of the images. Scale bar = 20 µm.
